# Hot-melt extruded ibuprofen ternary solid dispersions using in-line UV–Vis: Impact of an ionizable polymer on thermodynamics and dissolution

**DOI:** 10.1016/j.ijpx.2026.100536

**Published:** 2026-04-06

**Authors:** Matheus de Castro, Melissa Almeida, Christian Luebbert, Shadrack Joel Madu, Jatin Khurana, Mark Evans, Matthew Leivers, Gabriel Araujo, Mingzhong Li, Walkiria Schlindwein

**Affiliations:** a Leicester School of Pharmacy, De Montfort University, Leicester LE1 9BH, United Kingdom.; b Department of Pharmacy, School of Pharmaceutical Sciences, University of São Paulo, São Paulo, SP, Brazil.; c Amofor GmbH, Otto-Hahn-Str. 15, 44227 Dortmund, Germany; d Reckitt Benckiser, Dansom Lane, Hull HU8 7DS, United Kingdom.

**Keywords:** Amorphous solid dispersion, PC-SAFT, Thermodynamics, Dissolution, Stability

## Abstract

Amorphous solid dispersions (ASDs) remain a key strategy for enhancing the dissolution of poorly soluble APIs. Building on previous work with binary ibuprofen (IBU) blends, this study investigates the impact of incorporating poly(butyl methacrylate-*co*-(2-dimethylaminoethyl) methacrylate-co-methyl methacrylate), Eudragit EPO® (EPO), an ionizable third polymer into systems based on poly(vinylpyrrolidone-*co*-vinyl acetate), typically 60:40 VP:VA ratio, KOLVA64® (VA64), Polyvinylpyrrolidone, KOL17PF® (17PF) and hydroxypropyl methylcellulose acetate succinate, AQOAT AS-LMP (HPMCAS). Perturbed-Chain Statistical Associating Fluid Theory (PC-SAFT) modeling was employed to predict solid–liquid (SLE) and liquid-liquid (LLE) phase equilibria using binary interaction parameters (k_ij_). In comparison to Flory–Huggins's theory, PC-SAFT predicted broader metastable regions, corresponding to up to threefold higher achievable ibuprofen loadings. ASDs (35 wt%) with increasing EPO concentration (0–32.5 wt%) were successfully extruded using in-line UV–Vis spectroscopy for real-time monitoring, with samples grouped according to polymeric composition by principal component analysis (PCA). Solid-state analyses (FTIR, XRD, DSC) of extrudate samples confirmed no recrystallisation for up to six months (25 °C/70% RH). Small-scale DSC experiments within the PC-SAFT-predicted unstable zone confirmed crystallinity (95 wt% for VA64-EPO and 17PF-EPO; 50 wt% for HPMCAS-EPO). Dissolution studies under acidic conditions revealed complete release of blends with ≥20 wt% EPO within 5 min, outperforming binary formulations and maintaining supersaturation for hours. At pH 6.8, no significant dissolution improvement was seen, providing additional evidence of a diffusion-controlled release dependent on pH and API-polymer interactions. Overall, this work presents a novel PC-SAFT-based, predict-first approach to ternary ASD design, enabling higher drug loadings and controlled pH-responsive release.

## Graphical abstract

**Figure:**
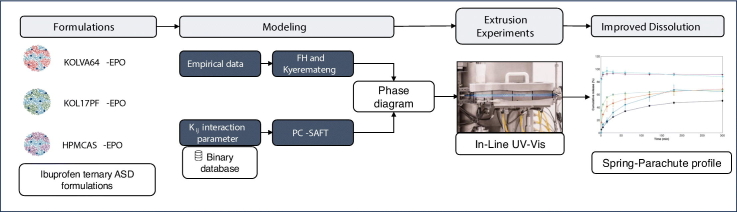

## 1 Introduction

Many modern active pharmaceutical ingredient (API) candidates exhibit poor aqueous solubility, creating an ongoing need for formulation strategies that can improve dissolution and enhance oral bioavailability. A common strategy to address this challenge is the formulation in the amorphous state, in which the API is converted into a higher-energy state with increased apparent solubility and the capacity to generate supersaturation upon dissolution (Li et al., 2019; Mantas et al., 2019). However, the thermodynamic instability inherent to amorphous materials poses a major limitation, as the API might recrystallize during storage or handling, compromising solubility, efficacy, and safety (Yu, 2001). Amorphous solid dispersions (ASDs) build upon this principle by molecularly dispersing the API within a miscible polymer matrix, preventing API nucleation and crystal growth, assuring the amorphous-state inherent solubility advantage.

Accurately predicting API–polymer miscibility remains central to the ASDs rational formulation design, as miscibility directly influences both solid-state stability and dissolution performance. Previous studies have demonstrated that the API solubility within a polymeric matrix can be estimated by examining the end-set temperatures of API–polymer blends of known composition (Tian et al., 2013). To date, Flory Huggins (FH) lattice theory is the more frequently used model to predict solid liquid (SLE) and liquid-liquid equilibrium (LLE) curves (Tian et al., 2013). Although less frequently employed, Kyeremateng empirical equation allows initial estimation of API solubility in polymer matrices and also provides risk zones during extrusion, useful to large-scale manufacturing (Kyeremateng et al., 2014). Perturbed-Chain Statistical Associating Fluid Theory (PC-SAFT) incorporates explicit contributions from molecular size, shape, dispersion forces and association effects, enabling a physics-based approach and is becoming frequently more utilised (Anderson, 2018; Pavliš et al., 2023; Prudic et al., 2014a). The T_g_ serves as an indicator of molecular mobility and thermodynamic stability in amorphous systems. In solid dispersions, the observation of a single T_g_  is typically interpreted as evidence of miscibility between the API and polymer. Empirical T_g_ models such as Gordon–Taylor and Kwei assume ideal volume additivity, so deviations from their predictions are interpreted as evidence of interactions (Pezzoli et al., 2018). To more effectively capture non-linear trends, Brostow–Kalogeras quadratic model provides a useful alternative (Kalogeras and Brostow, 2009), allowing additional insights about components interactions. The information obtained from these small-scale experiments is particularly valuable during formulation development for hot-melt extrusion (HME), where miscibility, melt rheology, and thermal behaviour strongly influence processability and final product performance.

Conventional ASDs typically rely on a single polymer to stabilise the API in its amorphous state, however, the incorporation of a third component has been increasingly explored as a strategy to further optimise the ASD performance (Baghel et al., 2016; Luebbert and Stoyanov, 2023; Trenkenschuh et al., 2024; Xie and Taylor, 2017). Surfactants have been shown to enhance supersaturation generation and dissolution rates (Guan et al., 2019), while also improving the physical stability of amorphous drugs by forming stronger intermolecular interactions and reducing recrystallisation tendency (Kapourani et al., 2021; Xie and Taylor, 2017). The inclusion excipients have also been reported to improve processability in HME, where modifications to the formulation can influence melt viscosity and extrusion behaviour (Trenkenschuh et al., 2024). Another valuable strategy relies on the addition of pH modifiers, such as sodium carbonate (Almotairy et al., 2021) and meglumine (Alhamhoom et al., 2024) to change dissolution microenvironment and improve pH-dependent dissolution.

Even though UV–Vis spectroscopy is generally less chemically specific than vibrational spectroscopic techniques such as Raman and NIR, it is more cost-effective and simpler to implement, particularly during early development phases (Mishra et al., 2025). In addition, the integration of multivariate data analysis has significantly expanded the applicability of UV–Vis, enabling the exploration of sample differences and pattern recognition, which is especially valuable for real-time process monitoring (Ríos-Reina and Azcarate, 2022). UV–Vis has been successfully applied to the evaluation of tablet critical quality attributes (Lillotte et al., 2021; Mészáros et al., 2020), cleaning monitoring and optimization (Steiner-Browne et al., 2024), and hot-melt extrusion (HME) monitoring (Bezerra et al., 2025; Schlindwein et al., 2018; Triboandas et al., 2024; Wesholowski et al., 2018). In this work, we aimed to assess UV–Vis as a tool for real-time monitoring and for identifying compositional patterns across different polymeric systems.

Ibuprofen (IBU) is a poorly soluble nonsteroidal anti-inflammatory drug that exhibits pH-dependent solubility due to its weakly acidic character (pKa ≈ 4.9). Eudragit® EPO (EPO), a cationic copolymer consisting of dimethylaminoethyl methacrylate, butyl methacrylate, and methyl methacrylate (2:1:1 ratio), is soluble below pH 5 and has been widely used to enhance supersaturation, improve dissolution, and act as a release modifier for ionisable APIs across various pH conditions (Darwich et al., 2024; Fine-Shamir and Dahan, 2019). This raises the relevant question of whether additional acid–base (ion-pairing) interactions between the carboxyl group of IBU and the tertiary amine functionalities of EPO could enhance physical stability or improve dissolution when incorporated into ASDs formulated with non-ionisable polymers.

Following our earlier investigation using IBU with Kollidon® VA64 (VA64), Kollidon® 17PF (17PF), and HPMCAS to produce binary ASD systems (De Castro et al., 2025), this study examines whether the addition of EPO offers any advantage in terms of stability or dissolution performance of extrudates. To this end, we apply the thermodynamic models described above to predict SLE, LLE, T_g_-based miscibility predictions, and we assess the dissolution behaviour of the resulting formulations under non-sink conditions in both acidic and basic pHs. This predict-first approach is further strengthened by the integration of In-line UV–Vis spectroscopy during extrusion, offering a novel, real-time validation of miscibility and establishing a direct link between thermodynamic predictions for ternary ASDs.

## 2 Materials

**Fig. 1:**
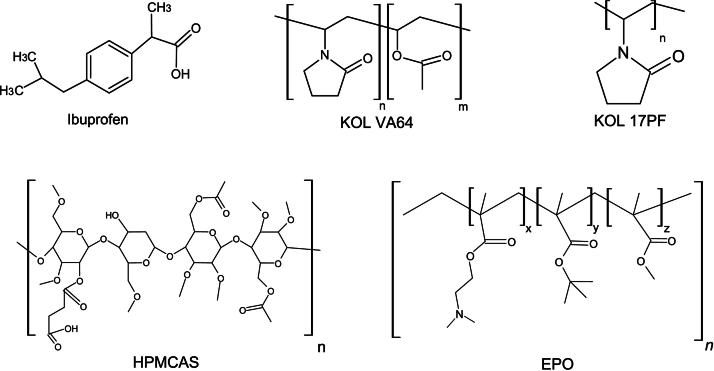
Ibuprofen (IBU), Kollidon® VA64 (VA64), Kollidon® 17PF (17PF), AQOAT AS-LMP (HPMCAS) and Eudragit EPO (EPO) molecular structures.

IBU, VA64, and 17PF were obtained from BASF (Ludwigshafen, Germany). HPMCAS was provided by Harke ChemLink (UK), and EPO by Evonik Pharma Polymers (Darmstadt, Germany). The API and polymers physicochemical properties are summarized in Table 1, and their chemical structures are shown in Fig. 1. True density (ρ), glass transition temperature (T_g_), melting temperature (T_m_), enthalpy of fusion (Δ_fus_H), and heat capacity change (ΔC_p_) were determined experimentally. (See Table 2.)

**Table 1:** Ibuprofen and polymers' true density and calorimetric properties.

Compound	*M*_w_ (g mol^−1^)	ρ (g cm^−3^)	*T*_g_ (°C)	*T*_m_ (°C)	Δ_fus_H(kJ mol^−1^)	Δ*C*_*p*_ (J mol^−1^ K^−1^)
IBU	206.28	1.11	−44.68	75.08	24.68	70.91
VA64	65,000	1.22	109.3	–	–	–
17PF	11,000	1.23	132	–	–	–
HPMCAS	18,000	1.30	122.5	–	–	–
EPO	47,000	1.14	57.2	–	–	–

**Table 2:** API and polymers PC-SAFT pure-component parameters.

Compound	*m*_i_	σ_i_ (Å)	ε_i_/*k* (K)	ε_i_^assoc^/*k* (K)	*k*_i_^assoc^	*N*_i_^assoc^
IBU ^a^	2.52	4.43	374.65	879.42	0.03	2/2
VA64 ^b^	2420.99	2.95	205.27	0.00	0.02	653/653
17PF ^c^	407.00	2.71	205.60	0.00	0.02	89/89
HPMCAS ^d^	895.70	2.91	316.87	2454.87	0.02	111/111
EPO ^e^	1645.00	3.74	258.89	0.00	0.02	848/848

Reference data: a – (Ruether and Sadowski, 2009), b – ((Luebbert et al., 2017b), c – (Prudic et al., 2014b), d – (Lehmkemper et al., 2017), e – amofor GmbH

## 3 Methods

### 3.1 Flory-Huggins (FH)

The end-set melting temperature of IBU was modelled using the FH approach (**Eq.** 1) to predict the solubility and miscibility limits in the polymer matrices. In this model, T_m_ represents the end-set melting temperature of pure IBU (K), and Δ_fus_H  its molar enthalpy of fusion. R is the universal gas constant, while $\phi_{\mathrm{API}}$ and $\phi_{\mathrm{polymer}}$  denote the volume fractions of the API and polymer, respectively. The API–polymer volume ratio (m) corresponds to the molar volume ratio of the polymer to the reference lattice site occupied by the API, and is calculated as the ratio of molecular volumes, derived from the molecular weight (M_w_) and true density (ρ) (**Eq.** 2). Densities were measured experimentally using a helium pycnometer (Micromeritics, US) equipped with a 3.5 cm^3^ cell operated under a 10-cycle protocol. The FH interaction parameter (χ) accounts for API–polymer interactions. Because χ varies with composition and temperature, it is commonly expressed as an entropic contribution (A) and a temperature-dependent enthalpic term (B) (**Eq.**3) (Tian et al., 2018). Here, we have made a similar assumption to Potter et al., 2018, treating ternary systems as pseudo-binary.

(1) $$\frac{1}{T}-\frac{1}{T_{m}}=-\frac{R}{\Delta_{\mathrm{fus}}H}\;\left[{\mathrm{ln\phi}}_{\mathrm{API}}+\left(1-\frac{1}{m}\right)\phi_{\mathrm{polymer}}+\chi \phi_{\mathrm{polymer}}^{2}\right]$$

(2) $$\begin{array}{c} m=\frac{N_{B}}{N_{A}}=\frac{\frac{M_{w\left(\mathrm{polymer}\right)}}{\rho_{\mathrm{polymer}}}}{\frac{M_{w\left(\mathrm{API}\right)}}{\rho_{\mathrm{API}}}}\; \end{array}$$

(3) $$\begin{array}{c} \chi =A+\frac{B}{T}\; \end{array}$$

The metastable and unstable regions can be calculated numerically solving the ${∆}_{\mathrm{mix}}G$ **(Eq.** 4**)** by $\Phi_{\mathrm{API}}$ and setting the first and second derivatives to 0 (**Eq.5** and **Eq.** 6).

(4) $$\begin{array}{c} {∆}_{\mathrm{mix}}G=\mathrm{RT}\left(\Phi_{\mathrm{API}}+\ln\Phi_{\mathrm{API}}+\frac{\Phi_{\mathrm{polymer}}}{m}\ln\Phi_{\mathrm{polymer}}+\chi \Phi_{\mathrm{API}}\Phi_{\mathrm{polymer}}\right)\; \end{array}$$

(5) $$\begin{array}{c} \left(\frac{\delta }{\delta {ɸ}_{\mathrm{API}}}\left(\frac{{∆}_{\mathrm{mix}}G}{\mathrm{RT}}\right)\right)=\ln{ɸ}_{\mathrm{API}}+1-\left(\frac{1}{m_{\mathrm{polymer}}}\right)-\left(\frac{1}{m_{\mathrm{polymer}}}\right)\ln\left(1-{ɸ}_{\mathrm{API}}\right)+\left(1-2{ɸ}_{\mathrm{API}}\right)\chi =0 \end{array}$$

(6) $$\begin{array}{c} \left(\frac{\delta^{2}}{\delta {ɸ}_{\mathrm{API}}^{2}}\left(\frac{{∆}_{\mathrm{mix}}G}{\mathrm{RT}}\right)\right)=\frac{1}{{ɸ}_{\mathrm{API}}}+\frac{1}{m_{\mathrm{polymer}}{ɸ}_{\mathrm{polymer}}}-2\chi =0 \end{array}$$

### 3.2 PC-SAFT

PC-SAFT is a thermodynamic equation of state that expresses the residual Helmholtz energy$\left(a^{\mathrm{res}}\right)$ as the sum of contributions from hard-chain repulsion (a^hc^), dispersion interactions (a^disp^ ), and specific association effects (a^assoc^  ) (**Eq.** 7) (Gross and Sadowski, 2001). This residual energy term is subsequently used to calculate the compressibility factor (Z) and the fugacity coefficient $\varphi_{i}^{L}$ (**Eq.** 8**–**9). PC-SAFT provides an explicit expression for the activity coefficient $\gamma_{i}$ , incorporating molecular size and shape, dispersion forces, and association interactions (**Eq.** 10).

(7) $$\begin{array}{c} a^{\mathrm{res}}=a^{\mathrm{hc}}+a^{\mathrm{disp}}+a^{\mathrm{assoc}}\; \end{array}$$

(8) $$\begin{array}{c} Z=1+\rho \left[\partial \left(\frac{a^{\mathrm{res}}/k_{b}\;T}{\partial \rho }\right)\right]\; \end{array}$$

(9) $$\begin{array}{c} \ln\varphi_{i}^{L}=\frac{\mu_{i}^{\mathrm{res}}}{K_{B}T}-\ln Z\; \end{array}$$

(10) $$\begin{array}{c} \gamma_{i}=\frac{\varphi_{i}^{L}}{\varphi_{0,i}^{L}}\; \end{array}$$

The mole fraction API solubility in a liquid amorphous phase is obtained by **Eq.**11, $\gamma_{\mathrm{API}}$ is the activity coefficient of the API in the mixture, $\Delta_{\mathrm{fus}}H$ is the API molar enthalpy of fusion, $\Delta_{\mathrm{fus}}C_{p}$ is the difference between the pure APIs' liquid and solid heat capacity.

(11) $$\begin{array}{c} x_{\mathrm{API}}=\frac{1}{\gamma_{\mathrm{API}}}\exp\left[-\frac{\Delta_{\mathrm{fus}}H}{\mathrm{RT}}\left(1-\frac{T}{T_{m}}\right)-\frac{\Delta_{\mathrm{fus}}C_{p}}{R}\left(1-\frac{T_{m}}{T}+\ln\frac{T_{m}}{T}\right)\right] \end{array}$$

In PC-SAFT, a binary interaction parameter, $k_{\mathrm{ij}}$ (**Eq.** 12) is introduced to account for non-ideal interactions between unlike components. The parameter modifies the cross-dispersive energy term in mixtures and is commonly adjusted to experimental phase equilibrium or melting point depression data to accurately describe intermolecular interactions. When amorphous–amorphous phase separation (AAPS) occurs, two coexisting phases, an API-poor phase (L^1^) and an API-rich phase (L^2^) are formed, each characterised by distinct molar fraction compositions x, as defined in **Eq.** 13 and **Eq.** 14. The equilibrium compositions of these two amorphous phases are obtained by simultaneously solving the equations at the temperatures of interest (Luebbert et al., 2017a).

(12) $$\begin{array}{c} k_{\mathrm{ij}}=k_{\mathrm{ij},\mathrm{int}}+k_{\mathrm{ij},\mathrm{slope}}\;T\left[K\right] \end{array}\;$$

(13) $$\begin{array}{c} x_{\mathrm{API}}^{L^{1}}\gamma_{\mathrm{API}}^{L^{1}}=x_{\mathrm{API}}^{L^{2}}\gamma_{\mathrm{API}}^{L^{2}} \end{array}$$

(14) $$\begin{array}{c} x_{\mathrm{polymer}}^{L^{1}}\gamma_{\mathrm{polymer}}^{L^{1}}=x_{\mathrm{polymer}}^{L^{2}}\gamma_{\mathrm{polymer}}^{L^{2}}\; \end{array}$$

Each associating compound requires the use of six parameters: the segment number (m_i_), segment diameter (σ_i_), the dispersion energy parameter (ε_i_/k_B_), association energy parameter (ε_i_^assoc^_/_k_B_), association volume (κ_i_^assoc^) and the number of association donor and acceptor sites (donors/acceptors – N_i_^assoc^) (Table 1). Binary interaction parameters ($k_{\mathrm{ij}}$) for IBU used in this work were taken from our previous study (De Castro et al., 2025) and are listed in Table 3.

**Table 3:** API and polymers PC-SAFT binary interaction parameters (k_ij_).

Compound	VA64	17PF	HPMCAS	EPO
Ibuprofen	0.05	0.06	0	0

### 3.3 Kyeremateng empirical equation

An empirical approach (**Eq.** 15) to determine the API–polymer solubility curve was developed by Kyeremateng et al. (2014) using only end-set temperatures obtained by DSC. Here, T represents the API solubility temperature, Tₘ is the melting point of the pure API, $x_{\mathrm{API}}$ is the API content in the mixture, and A, b, and C are fitting parameters. In this work, we fixed C to 0 and optimized A and b, followed by C optimization as already reported in literature (Mathers et al., 2021).

(15) $$\begin{array}{c} T=-Ae^{bx_{\mathrm{API}}}+T_{m}+C\; \end{array}$$

### 3.4 Gordon-Taylor, Kwei and Brostow-Kalogeras models

The glass transition behaviour of the blends was modelled as a function of API content using the GT, Kwei, and BK equations (Han et al., 2019). The GT constants $K_{1}$ and $K_{2}$  were obtained by fitting the experimental T_g_ data (**Eq.** 16).

(16) $$\begin{array}{c} T_{g}=\frac{w_{\mathrm{API}}T_{g,\mathrm{API}}+K_{1}w_{\mathrm{pol}1}T_{g,\mathrm{pol}1}+K_{2}w_{\mathrm{API}}T_{g,\mathrm{pol}2}}{w_{\mathrm{API}}+K_{1}w_{\mathrm{pol}1}+K_{2}w_{\mathrm{pol}2}}\; \end{array}$$

For the Kwei model (**Eq.** 17), the calculated $K_{1}$ and $K_{2}$  values were applied, while the additional parameters q1 and q2   were determined through data fitting (Kwei, 1984).

(17) $$\begin{array}{c} \;T_{g}=\frac{w_{\mathrm{API}}T_{g,\mathrm{API}}+K_{1}w_{\mathrm{pol}1}T_{g,\mathrm{pol}1}+K_{2}w_{\mathrm{API}}T_{g,\mathrm{pol}2}}{w_{\mathrm{API}}+K_{1}w_{\mathrm{pol}1}+K_{2}w_{\mathrm{pol}2}}+q_{w_{\mathrm{API}}\;w_{\mathrm{pol}1}}+q_{w_{\mathrm{API}}\;w_{\mathrm{pol}2}} \end{array}$$

To account for non-ideal mixing effects, the experimental data were also fitted using the BK model, as proposed by Kalogeras and Brostow (2009), which provides an alternative approach to T_g_ shifts using a quadratic equation.

(18) $$\begin{array}{c} ∆T_{g}=wT_{g1}+\left(1-w_{1}\right)T_{g2}+w_{1}\;\left(1-w_{1}\right)\left[a_{0}+a_{1}\;\left(2w_{1}-1\right)+a_{2}\;{\left(2w_{1}-1\right)}^{2}\right]\; \end{array}$$

### 3.5 Model accuracy evaluation

Model accuracy evaluation was performed through average absolute relative deviation (AARD) and absolute relative deviation (ARD), as per equations below (**Eq.** 19 and **Eq.** 20).

(19) $$\begin{array}{c} \mathrm{AARD}=\frac{1}{N}\;\sum_{i=1}^{N}\left|\frac{T_{\mathrm{measured}}-T_{\mathrm{predicted}}}{T_{\mathrm{measured}}}\right|\;X\;100\; \end{array}$$

(20) $$\begin{array}{c} \mathrm{ARD}=\frac{1}{N}\;\sum_{i=1}^{N}\left(\frac{T_{\mathrm{measured}}-T_{\mathrm{predicted}}}{T_{\mathrm{measured}}}\right)\;X\;100\; \end{array}$$

### 3.6 Differential Scanning Calorimetry (DSC)

API–polymers mixtures studied (90–20% *w*/w) were obtained by weighing and mixing using pestle and mortar for 10 min, to avoid unwanted API amorphization. Differential scanning calorimetry was performed in triplicate using a DSC TA25 (Waters, USA) equipped with an RCS90 cooling system to determine T_m_ and T_g_ for neat IBU and blends. The instrument was purged with nitrogen at 50 mL/min. Samples underwent a heat–cool–heat protocol: an initial heating from 0 to 100 °C at 1 °C/min, quenching to −90 °C at 10 °C/min, and a second heating from 0 to 100 °C at the same rate for T_g_ determination. Melting depression data (MPD) was used to in the FH and the Kyeremateng models whilst T_g_ data used in GT, Kwei and BK models (MATLAB® vR2024b). The same protocol was used to evaluate milled extrudates.

### 3.7 Hot-melt extrusion

Physical mixtures were prepared by blending IBU and the respective polymers (Table 4) using a Turbula mixer T2F (Willy A. Bachofen, Switzerland) for 10 min to ensure homogeneous distribution of the components. The blends were subsequently processed using a Leistritz Nano-16 (Somerville, NJ, USA) twin-screw extruder equipped with 16 mm co-rotating screws with conveying and kneading elements. For all experiments, the screw speed was fixed at 100 rpm, and the barrel temperature profile was set to increase gradually from 60 °C to 80 °C within 4 distinct barrel zones. Extrudate strands were milled into powder using a ball mill (MM200, Retsch, Germany) operating at 22 rpm for 2 min, and the resulting material was used for subsequent physical and analytical characterization.

**Table 4:** Formulations extruded containing 35% IBU and their respective code.

Formulation code	VA64 (wt%)	17PF (wt%)	EPO (wt%)
VA64-EPO-0	65	–	0
VA64-EPO-5	60	–	5
VA64-EPO-10	55	–	10
VA64-EPO-20	45	–	20
VA64-EPO-32.5	32.5	–	32.5
17PF-EPO-0	–	65	0
17PF-EPO-5	–	60	5
17PF-EPO-10	–	55	10
17PF-EPO-20	–	45	20
17PF-EPO-32.5	–	32.5	32.5

### 3.8 In-line UV-Vis spectroscopy

For the in-line analysis, an Inspectro X UV–Vis spectrophotometer (ColVisTec, Berlin, Germany) was connected directly to the extruder die. Measurements were performed in transmission mode using two TPMP probes (ColVisTec, Berlin, Germany), covering a wavelength range from 220 to 800 nm at a sampling frequency of 0.5 Hz. The probe is equipped with a sapphire window and detachable fiber optics to facilitate calibration.

After spectral acquisition, noise reduction was achieved by selecting the most informative wavelength region between 220 and 350 nm. Several preprocessing techniques were evaluated, and standard normal variate (SNV) and Savitzky-Golay second derivative were identified as the most suitable method based on the variance explained in the resulting models. Finally, the data were mean centered prior to multivariate analysis.

### 3.9 Fourier Transformed infrared Spectroscopy (FTIR)

FTIR was used to identify interactions between IBU and the polymers. Spectra of API-polymers physical mixtures, and extrudates were collected using an Alpha-Platinum spectrophotometer (Bruker, DE) equipped with a diamond ATR accessory. All measurements were recorded at room temperature over 4000–600 cm^−1^ with a resolution of 2 cm^−1^ and 128 scans. Spectra were collected and pre-processed (baseline corrected and smoothed with 25 points) using Opus 7.5 software.

### 3.10 Powder X-ray Diffraction (PXRD)

PXRD was used to assess the solid-state form of IBU in physical mixtures and extruded samples. Diffraction patterns were collected using a D2 Phaser X-ray diffractometer (Bruker, DE). Measurements were recorded from 3 to 40° 2θ with a step size of 0.02° and a step time of 0.5 s, operating at 10 mA and 30 kV. Samples were mounted on a rotating holder set at 5°/min.

### 3.11 Stability studies

Extrudate samples were stored at 25 °C/70% RH, and their physical stability assessed at three and six months by DSC, XRD, and FTIR. In parallel, to validate PC-SAFT predictions within the unstable zone, small-scale samples were prepared directly in DSC pans at drug loadings exceeding the predicted miscibility limit for each ternary system (IBU/VA64-EPO 95:5 wt%; IBU/17PF-EPO 95:5 wt%; IBU/HPMCAS-EPO 50:50 wt%) and evaluated for crystallinity over the same storage period by DSC. The high drug loading of 95 wt% IBU selected for VA64-EPO and 17PF-EPO systems reflects their broader predicted miscibility window, whereas the lower loading of 50 wt% was required for HPMCAS-EPO due to its comparatively poorer miscibility with IBU. These samples were prepared using a DSC heating run from 0 to 100 °C at 1 °C/min and analyzed using the same range at 10 °C/min.

### 3.12 Non-sink dissolution experiments

IBU equilibrium solubility measurements were first conducted in acidic (pH 2.0, 0.02 M HCl) and basic (pH 6.8 phosphate buffer) media using the shake-flask method in triplicate. Excess IBU was added to 75 mL of each medium and agitated at 75 rpm for 24 h at 37 °C. Following equilibration, samples were centrifuged at 12,000 rpm for 1 min, and the supernatant was appropriately diluted. Quantification was performed by HPLC (Hewlett Packard 1100) using a Hypersil BDS C18 column (ThermoFisher), with a mobile phase of acetonitrile–water (0.1% TFA) at 0.8 mL/min and UV detection at 264 nm. The equilibrium solubilities obtained were 61.6 ± 0.35 μg/mL at pH 2.0 and 682 ± 14.04 μg/mL at pH 6.8.

Based on these solubility figures, dissolution testing was carried out under non-sink conditions using a sink index (SI) of 0.5 (**Eq.**21) using milled extrudates. Experiments were performed in triplicate using an Erweka DT Light apparatus (paddle method) at 37 °C and 75 rpm in 100 mL vessels containing either pH 2.0 or pH 6.8 media. 2 mL aliquots were withdrawn at defined time points (1, 5, 15, 30, 60, 120, 180, and 300 min), centrifuged at 12,000 rpm for 1 min, and immediately replaced with fresh medium to maintain constant volume. The supernatant was analyzed using the same validated HPLC method described above.

(21) $$\begin{array}{c} \mathrm{SI}=\frac{C_{s}V}{D}\; \end{array}$$

## 4 Results

### 4.1 DSC experiments

#### 4.1.1 Melting point depression

**Fig. 2:**
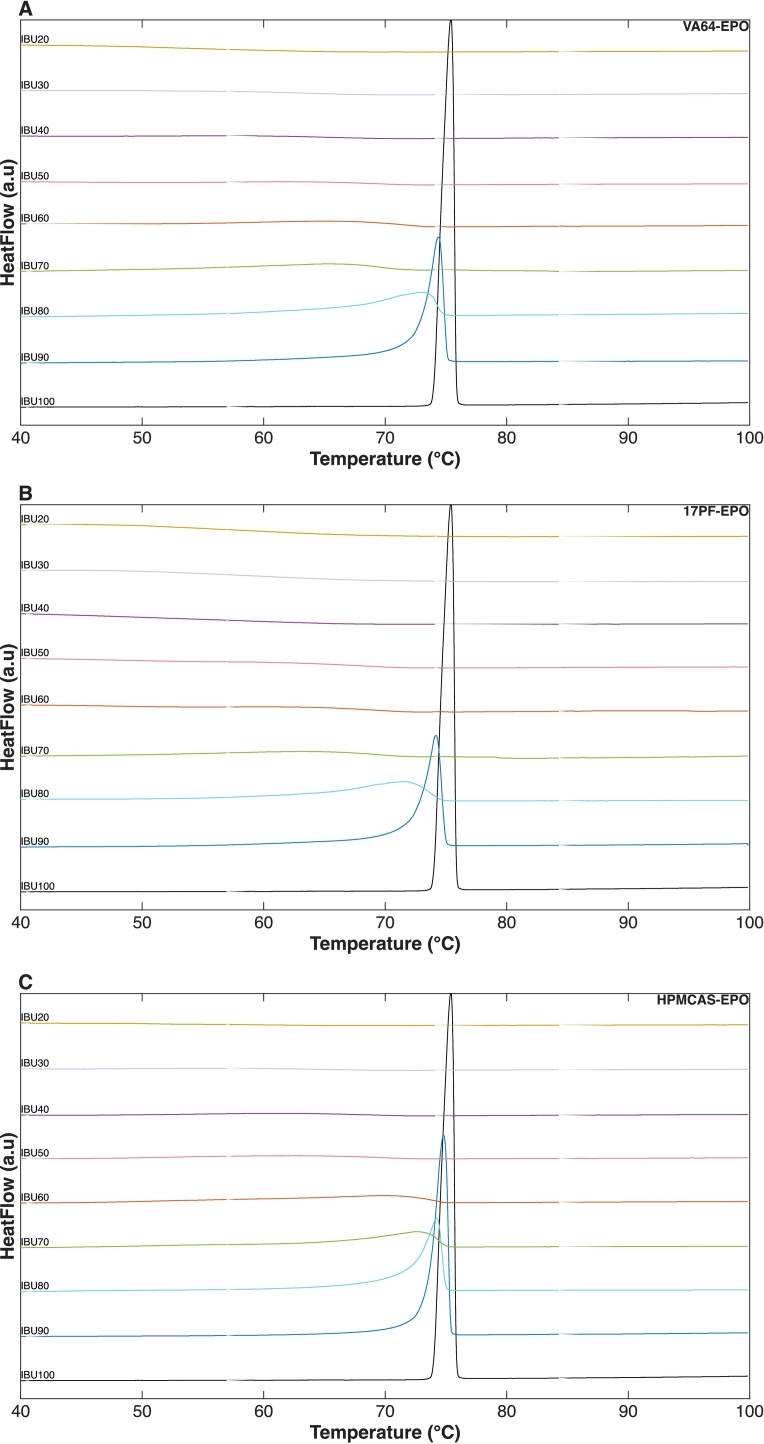
DSC thermograms of different IBU-polymer compositions, showing melting point depression (1 °C/min) during first cycle. A) VA64-EPO. B) 17PF-EPO. C) HPMCAS-EPO.

The samples first DSC scan with varying API concentrations (90–20 wt%) are shown in Fig. 2. The presence of molecular interactions between the API and the polymer leads to strong intermolecular affinity, which lowers the API activity coefficient in the mixture. As described by Eq. 11, this reduction results in an increased solubility of the API in the polymer matrix and manifests experimentally as enhanced MPD. Consistent with earlier findings (De Castro et al., 2025), 17PF–EPO polymeric blend exhibited higher MPD compared to VA64–EPO blends, indicating stronger molecular interactions within the drug (Browne et al., 2020). In contrast, minimal end-set depression was observed for the HPMCAS–EPO system at polymer loadings below 40 wt%, reflecting weaker IBU-HPMCAS interactions (Iemtsev et al., 2020). No endothermic melting peak characteristic of crystalline IBU was detected during the second heating cycle for any of the samples indicating that samples remained completely amorphous during cooling/second heating (**Fig. S1**).

Compared to the binary API- single polymer ASDs (De Castro et al., 2025), it was initially unclear whether blends containing polymer mixtures would exhibit a synergistic API–polymer interaction effect, further reducing the melting point end-set. As shown in **Fig. S2**, although the ternary average end-set values tend to be lower, no statistical difference was observed between the binary and ternary systems for VA64 and 17PF, whereas minor differences were detected for HPMCAS. Similar observations were reported by Potter et al. (2018) for an ibuprofen–HPMCP–Soluplus system, where the incorporation of a low-T_g_ polymer resulted in limited additional melting point depression compared to binary blends. These findings were attributed to competing effects, where a reduction in melt viscosity—associated with the T_g_ of Soluplus—did not translate into stronger API–polymer interactions. An analogous situation is present in the current study, where high-T_g_ polymers (VA64, 17PF, or HPMCAS) are combined with the low-T_g_ polymer EPO. While the addition of EPO is expected to reduce melt viscosity and increase molecular mobility, this effect alone does not necessarily enhance specific API–polymer interactions or further lower the melting end-set. Furthermore, molecular modeling studies have demonstrated that proton-donating groups of Ibuprofen involved in hydrogen bonding may preferentially interact with the polymer exhibiting the highest interaction affinity, leading to localized interaction domains or “interaction pockets” (Saal et al., 2017; Yani et al., 2017). These preferential interactions can limit the effective contribution of additional polymers, thereby constraining further melting point depression despite changes in rheological properties.

#### 4.1.2 Glass transition

**Fig. 3:**
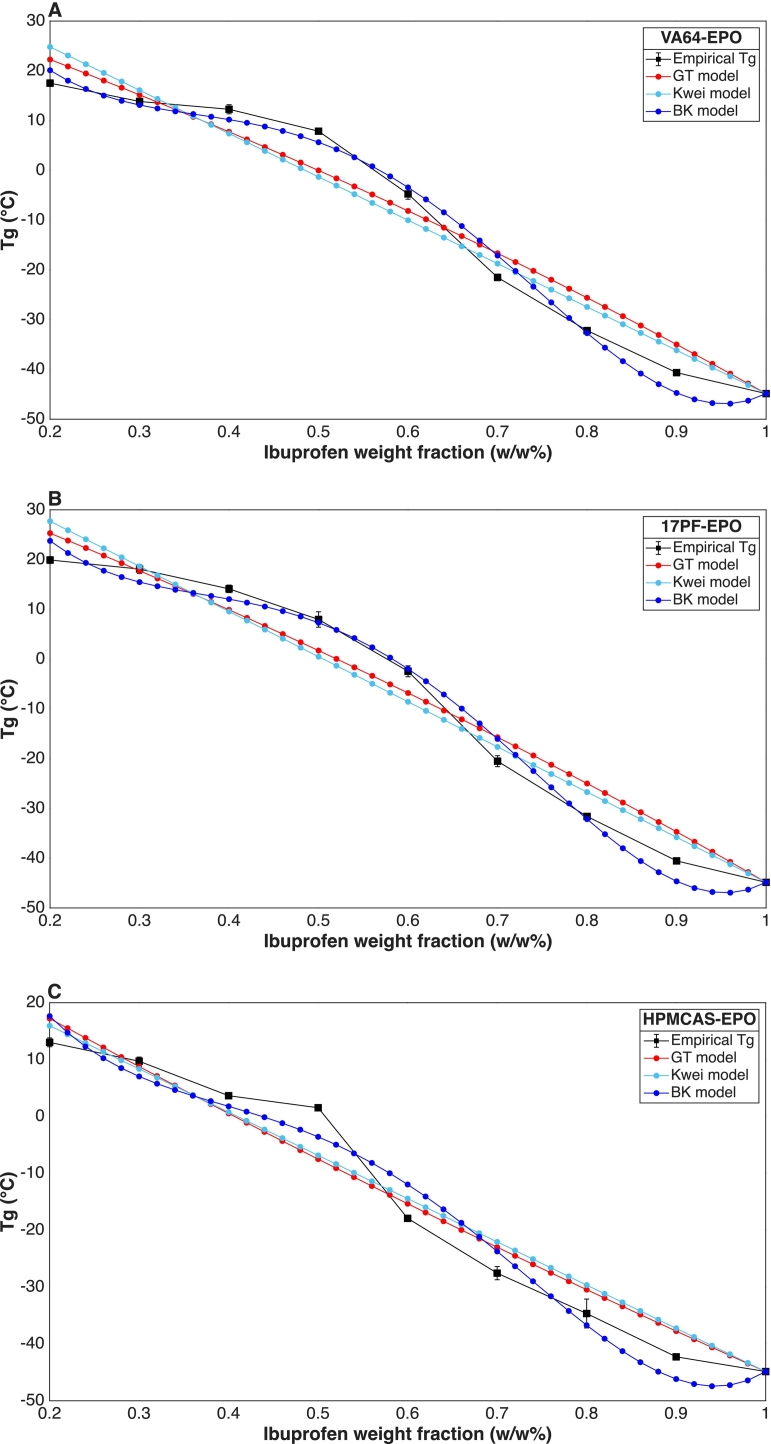
T_g_ modeling using GT and Kwei and BK equations for IBU and A) VA64-EPO. B)17PF-EPO. C) HPMCAS-EPO.

As shown in Fig. 3**,** there is an inverse trend between API loading and empirical T_g_ values across all samples tested, consistent with the strong IBU plasticizing effect (T_g_ = −44.2C), resulting on a sigmoidal curve. This trend has also been reported for binary IBU–EPO blends with various polymers (Tian et al., 2020) and contrasts with the monotonic behaviour observed for binary IBU–VA64 and IBU–17PF systems (De Castro et al., 2025), indicating an additional influence of EPO in the ternary blends during the second DSC heating (**Fig. S3**). This T_g_ modulation may arise possibly from additional ionic-ionic or dipole-ionic interactions between IBU–EPO enabled at molten state with increased mobility (Biedrzycka and Marcinkowska, 2023; Patel and Minko, 2025).

Positive and negative deviations from ideal behaviour were observed across the blends in this study. Such deviations typically arise from non-ideal mixing, where differences in free volume, molecular weight, or cohesive energy density between components produce T_g_ shifts that diverge from ideal predictions (Nair et al., 2001). Positive deviations generally indicate stronger API–polymer interactions, whereas negative deviations imply that API–polymer interactions are weaker than API-API interactions (Cools and Van Den Mooter, 2025).

Considering this non-monotonic T_g_ trend, BK model showed superior fitting accuracy (R^2^ > 0.98) modeling non-linear data as reported for other API-polymer systems (Kalogeras, 2010). The modeling parameter a_0_  could reflect the overall strength of intermolecular interactions between blend components. Negative a_0_ values are often associated with weaker API–polymer interactions and potential API self-association, as observed for indomethacin–PVPVA systems, despite evidence of hydrogen bonding with this polymer (Kalogeras, 2010; Pezzoli et al., 2018). Even though all blends have a_0_  negative values (**Table S1**), HPMCAS–EPO system exhibited the lowest figure (a_0_ = −103.6), which would support this interpretation, but these would contradict for the other blends (VA64-EPO a_0_ = −53.67; 17PF-EPO a_0_ = −70.03). The a₂ parameter captures higher-order non-idealities, including interaction strength and free-volume changes (Patel and Minko, 2025). While the fitted a₂ values are similar in magnitude across the blends, the slightly more negative value obtained for IBU–KOL17–EPO (a₂ = −309.2) and IBU-VA64-EPO (a₂ = −291.3) suggests stronger overall interaction-driven deviations from ideal mixing. In contrast, the less negative *a₂* observed for IBU–HPMCAS–EPO (a₂ = −265.2) is consistent with a system exhibiting weaker interactions.

#### 4.1.3 Phase diagram modeling

**Fig. 4:**
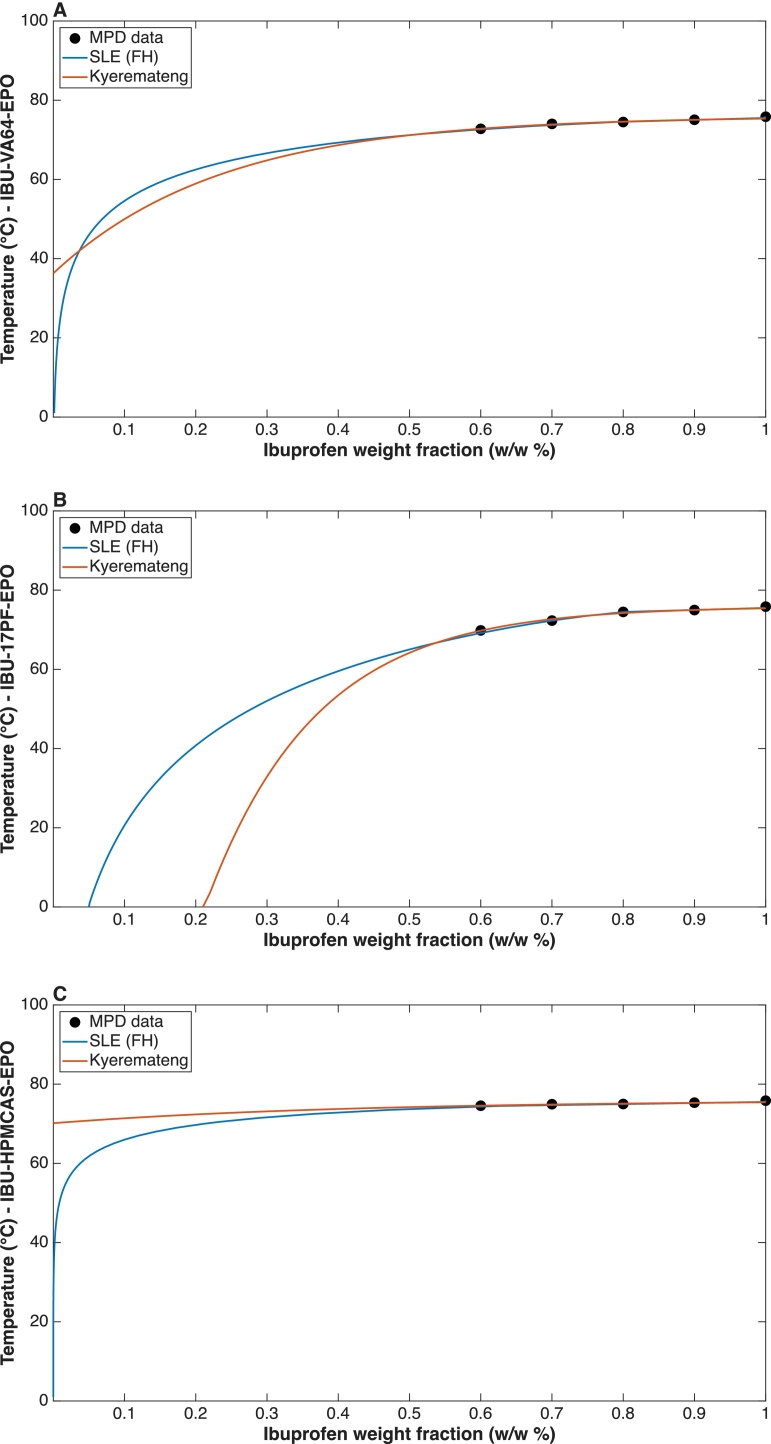
Phase diagrams of ternary blends comparing MPD data and SLE modelled using FH and Kyeremateng empirical equation A) VA64-EPO, B) 17PF-EPO, C) HPMCAS-EPO.

**Fig. 5:**
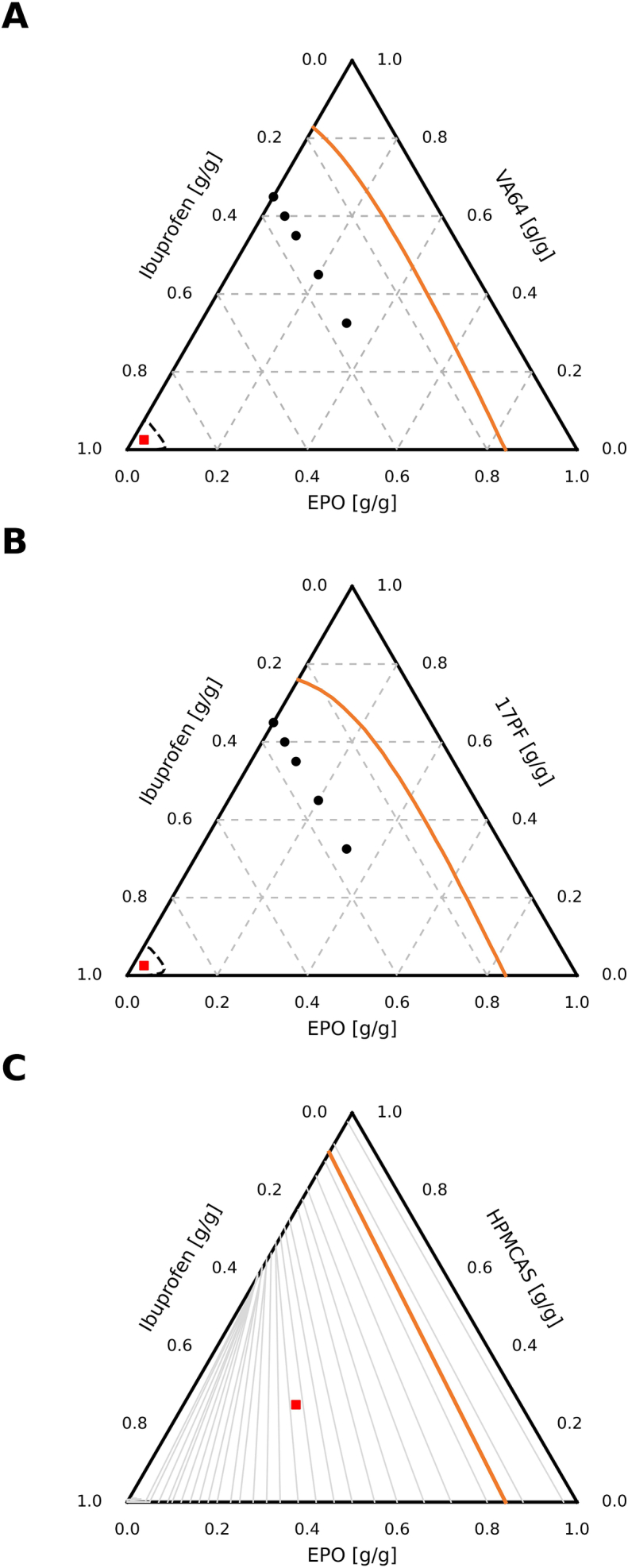
PC-SAFT SLE and LLE predictions for at 25 °C: A) IBU-VA64-EPO. B) IBU-17PF-EPO. The orange line predicts IBU solubility in the matrices, where the dashed black line is the spinodal curve. C) IBU-HPMCAS-EPO, the grey lines indicate the tie lines, the orange line is the predicted IBU solubility in the HPMCAS-EPO mixture (metastable). Owing to the extensive overlapping demixing region, the entire ternary composition space is immiscible, and the tie lines span the full ternary diagram. Black circles (•) correspond to extrudate samples whilst red square () shows the small-scale stability experiments. (For interpretation of the references to colour in this figure legend, the reader is referred to the web version of this article.)

Fig. 4 and Fig. 5 present the phase diagrams of the three API–polymer ternary systems, constructed from experimentally determined MPD data using FH, Kyeremateng and PC-SAFT models. The SLE curves indicate that above ∼70 °C all compositions lie within the single-phase region, suggesting that every system can be processed as a homogeneous melt across a typical HME temperature range, independent of API loading (Moseson and Taylor, 2018). Although the models predict different absolute solubility limits based on their different principles, they consistently show that HPMCAS-based matrices possess the lowest solubilizing capacity, whereas the other polymers provide comparatively higher drug solubility. In the HPMCAS–EPO system, high temperatures are still required to process low API loadings, a similar pattern to the high-risk zone within the recently described HME risk classification system (HCS) by Kyeremateng et al. (2022), thus it would not be an initial blend of choice. Liquid–liquid equilibria were approximated using FH interaction parameters obtained via a mixing rule from the pure polymers; although a rigorous treatment would require the application of cloud- and shadow-curve concepts for such pseudo binary sections, this was not pursued here for reasons of simplicity, and an analogous cloud/shadow analysis was likewise not performed for the PC-SAFT calculations.

Following the analysis of the ternary phase behaviour using FH theory and the Kyeremateng approach (Fig. 4), PC-SAFT was subsequently applied to reassess the ternary phase diagrams of the IBU–polymer–EPO systems shown in Fig. 5. In contrast to the lattice-based and empirical model, PC-SAFT provides a molecular-based description of the ternary solubility and miscibility landscape, accounting explicitly for component size, dispersion interactions and specific intermolecular effects.

In the ibuprofen–VA64–EPO system (Fig. 5A), PC-SAFT predicts a solubility approximately constant at around 18 wt% across the entire VA64–EPO composition range, indicating that the addition of EPO does not significantly enhance drug solubility in this ternary system. This value is significantly higher than the value predicted via FH. No liquid–liquid equilibrium is predicted within the investigated composition space, suggesting full miscibility of IBU with the VA64–EPO polymer matrix and the absence of relevant amorphous–amorphous phase separation.

An analogous behaviour is observed for the IBU–17PF–EPO system (Fig. 5B). Here again, PC-SAFT predicts complete miscibility of the polymeric components and a single crystalline solubility boundary for IBU. The lack of tie lines or demixing regions indicates that amorphous phase separation is thermodynamically unfavorable over a wide composition range, and that the ternary system is a fully miscible polymer blend. In contrast, the IBU–HPMCAS–EPO system (Fig. 5C) exhibits a fundamentally different phase behaviour: PC-SAFT predicts a pronounced immiscibility gap originating from the binary HPMCAS–EPO binary side on the right side. This binary polymer–polymer immiscibility propagates into the ternary phase diagram, resulting in extensive liquid–liquid equilibrium regions characterised by tie lines spanning a large fraction of the composition space. Consequently, the system is thermodynamically driven toward phase separation irrespective of the IBU content. PC-SAFT predictions indicate that the VA64–EPO and 17PF–EPO mixtures form fully miscible ternary blends with a well-defined IBU solubility limit of 18 wt%, whereas the HPMCAS–EPO system is dominated by the polymer–polymer immiscibility. Based on this evidence we chose to use 35 wt% IBU loading in the extrudates VA64 and 17PF based extrudates to challenge the higher metastable region predicted by PC-SAFT.

### 4.2 Extrudates analysis

#### 4.2.1 In-line UV–Vis

A preliminary visual inspection of the raw UV–Vis spectra revealed highly similar spectral profiles across all formulations. The main differences were observed in absorbance intensity rather than in overall spectral shape, with VA64-EPO-0 exhibiting the lowest intensity and the remaining formulations showing only subtle variations. Notably, no abrupt baseline shifts, typically associated with API–matrix insolubility (Schlindwein et al., 2018; Wesholowski et al., 2018) were detected. This observation provides preliminary experimental support for the PC-SAFT predictions of blends' phase diagram.

**Fig. 6:**
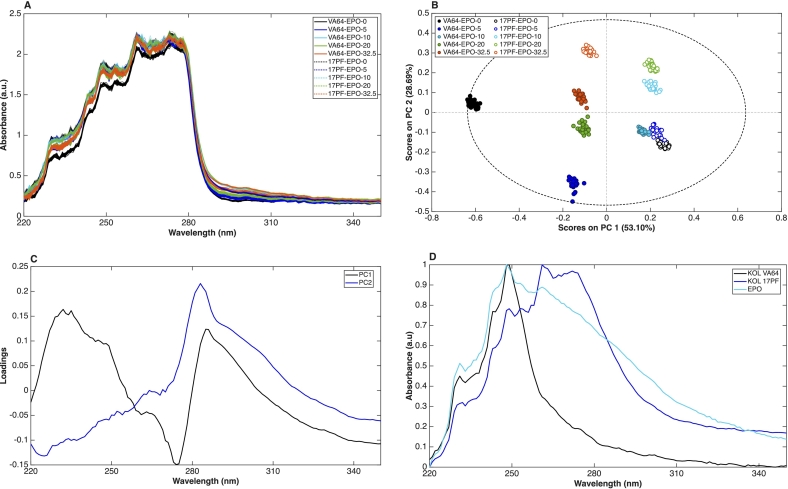
A) UV–Vis raw spectra, B) PCA1 and PC2 scores plot, C) PC1 and PC2 loadings plot and D) Normalized polymers UV–Vis spectra.

The PCA scores plot (Fig. 6B) shows that formulations without EPO (VA64-EPO-0 and 17PF-EPO-0) cluster on opposite sides of PC1, which explains 53.10% of the total variance. This separation suggests that PC1 primarily reflects differences in polymer type. The same pattern is observed for other samples in which VA64-based systems are predominantly located at negative PC1 scores, whereas 17PF formulations are distributed on the positive side. Examination of the PC1 loadings (Fig. 6C) indicates that this discrimination is mainly driven by spectral differences in the regions 230–250 nm, 270–275 nm, and 280–290 nm (Fig. 6D). Despite considerable spectral overlap, analysis of the individual polymer spectra shows that the positive loading around 280–290 nm corresponds to the broader and more intense absorption band characteristic of 17PF. In contrast, VA64 exhibits relatively stronger contributions at lower wavelengths and a sharper decline in absorbance beyond approximately 260 nm. Interestingly, VA64-EPO-10 and 17PF-EPO-32.5 deviate from the general PC1 trend, appearing on the opposite side of the expected clustering. This behaviour could reflect the non-linear interaction trends between components seen in the Tg curves.

In contrast, PC2 (28.69%) is mainly associated with variations in EPO concentration (Fig. 6B). For VA64-based blends, a progressive shift toward higher PC2 scores is observed with increasing EPO content, indicating a systematic compositional effect. In contrast, the separation between 17PF-EPO-0 and 17PF-EPO-5 is subtle, becoming more pronounced only at higher EPO concentrations. The corresponding PC2 loadings (Fig. 6C) display a strong positive contribution in the 280–290 nm region, consistent with the broad absorbance band observed in the EPO spectrum. While previous studies have identified drug loading as the primary source of variability in ASD systems (Bezerra et al., 2025; Wesholowski et al., 2018), the present findings demonstrate that changes in polymer composition at fixed drug loading can also be detected using in-line UV–Vis spectroscopy, supporting its application as a process analytical technology (PAT) tool.

#### 4.2.2 Fourier Transformed infrared Spectroscopy (FTIR)

FTIR spectroscopy was applied to evaluate API–polymer interactions across all extrudates and to determine the solid-state form of IBU. Such interactions are typically seen as shifts in characteristic vibrational bands from individual components. IBU exhibits a pronounced carbonyl stretching vibration at 1705 cm^−1^, C—H stretching near 3100 cm^−1^, two well-defined bands at 2731 and 2633 cm^−1^ that could reflect API dimer formation, and an aromatic vibration at 778 cm^−1^ (Yu et al., 2009). VA64 is characterised by two carbonyl bands from the vinyl acetate (1732 cm^−1^) and pyrrolidone (1654 cm^−1^), and an acetate C—O stretching near 1230 cm^−1^ (Hurley et al., 2019). 17PF exhibits a single carbonyl stretching band at ∼1650 cm^−1^ corresponding to the pyrrolidone group. Eudragit EPO presents an ester carbonyl band at ∼1723 cm^−1^ together with dimethylamine-associated vibrations at 2822 and 2767 cm^−1^ (Nyamba et al., 2023).

**Fig. 7:**
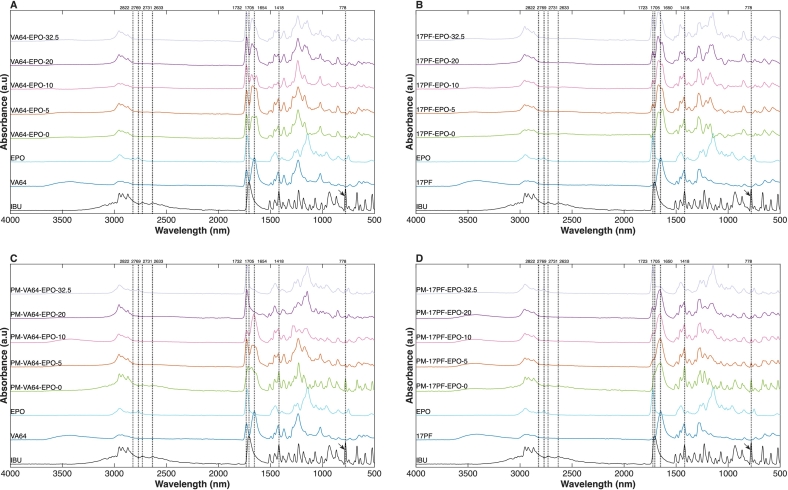
FTIR spectra from: A) VA64-EPO EXT, B) 17PF-EPO EXT, C) VA64-EPO PM, D)17PF-EPO PM. The arrow indicates the band at 778 nm. Dotted lines are guidelines for the described wavelengths.

In the VA64-based blends (Fig. 7A), the binary sample (VA64–EPO-0) shows two distinct peaks in the pyrrolidone carbonyl region (∼1655 cm^−1^), reflecting a substantial shift of the IBU carbonyl band, consistent with its favorable interactions with pyrrolidone rather than acetate moieties, as supported by molecular modeling (Yani et al., 2017). With increasing EPO content this region becomes progressively broadened and the separation between the two peaks becomes less clear. The band near 1730 cm^−1^ probably results from the overlapping carbonyl contributions from EPO esters and polyvinyl acetate units in VA64, therefore no definitive conclusion from EPO's role in the interaction can be derived based on this region alone.

A similar trend is seen in the 17PF-based blends (Fig. 7B), where the IBU carbonyl peak shifts to ∼1660 cm^−1^ due to interactions with pyrrolidone groups. For these blends, the EPO carbonyl band increases proportionally with its concentration and exhibits a minor shift toward ∼1725 cm^−1^ possibly indicating an interaction degree. For both VA64 and 17PF blends (Fig. 7A**/B**), the characteristic EPO dimethylamine bands at 2822 and 2769 cm^−1^ are absent, likely due to their involvement in intermolecular interactions with acidic IBU (Biedrzycka and Marcinkowska, 2023).

Examining the blends physical mixtures (Fig. 7C**/D**), a similar IBU carbonyl peak shift is observed resulting on a convoluted carbonyl region (1700–1600 cm^−1^). These spectral changes suggest the presence of solid-state API–polymer interactions, that have previously been reported for PVP–IBU systems (Sekizaki et al., 1995). This interpretation is justified considering that VA64 is a copolymer comprised of vinylpyrrolidone and vinyl acetate in a 6:4 ratio. Furthermore, considering that the formulations in this study use relatively high polymer-API ratio (2:1), the combination of hydrogen bonding and substantial polymer content would be expected to influence the carbonyl region significantly. Differently from extrudates, for high EPO content samples (32.5%) unaltered dimethylamine bands (2822 and 2769 cm^−1^) can be seen, suggesting that only extrudate samples can form ionic bonds with IBU.

In the context of ASDs is important to probe amorphization and the peak at 778 cm^−1^ can be considered a unique marker of crystalline IBU (Mužík et al., 2020). This peak is absent in extrudate samples from both polymeric matrices (Fig. 7A**/B)** whereas is evident in PMs (Fig. 7C**/D and Fig. S4** offer a focused graph on this peak), indicating that although solid-state interactions between API-polymers occur, they are insufficient to disrupt the crystalline structure or induce amorphization without the shear and thermal input provided during extrusion. Once amorphized, the samples depict early-stage stability, with no detectable reappearance of crystalline signatures. After three and six months, the extrudates retained an unchanged spectral profile, indicating that the amorphous state remained stable with no evidence of recrystallization over the study period (**Fig. S5 A-D** and **Fig. S6 A-D**).

#### 4.2.3 Powder X-ray Diffraction (PXRD)

PXRD analysis was used to evaluate the solid-state form of IBU within the amorphous solid dispersions. Neat crystalline IBU presents distinct diffraction peaks at 6.001°, 16.52°, and 22.208° 2θ (Mallick et al., 2008), which were used as markers to detect any remaining crystalline drug. The polymeric carriers used in this study are amorphous and thus exhibit only broad halo patterns (**Fig. S7**).

**Fig. 8:**
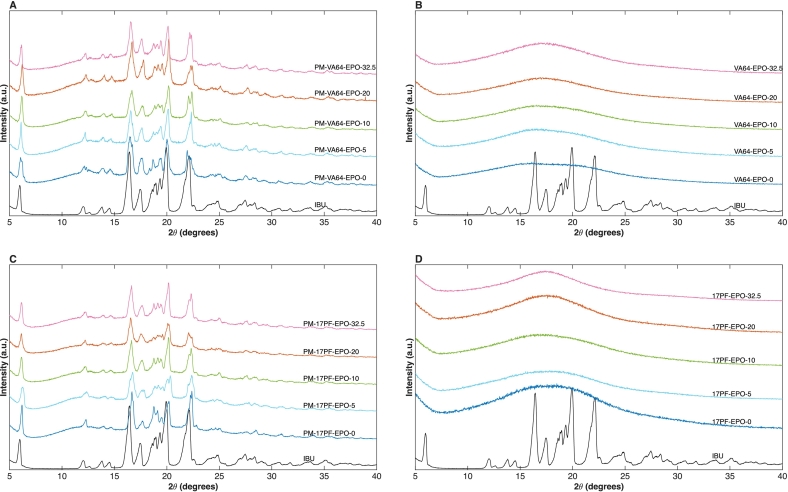
PXRD diffractograms from: A) VA64-EPO PM, B) VA64-EPO EXT, C) 17PF-EPO PM, D) 17PF-EPO EXT.

As shown in Fig. 8 **A/C**, the characteristic IBU peaks remain clearly visible in the physical mixtures, demonstrating that the drug persists in a highly ordered crystalline state regardless of the polymer combination and that any solid-state interactions seen at this stage are insufficient to disrupt its structure. In contrast, the extruded samples (Fig. 8B**/D**) display a complete loss of these diffraction peaks across all binary and ternary formulations, instead presenting a broad amorphous halo typical of the polymer matrices (Ponnammal et al., 2018). The same halo pattern was observed after three and six months in the stability samples, indicating that no recrystallisation had occurred over the study period (**Fig. S8 A/B** and **Fig. S9 A/B**). These results are consistent with the FTIR data and with the absence of IBU melting endotherms in DSC curves and further support the use of the 778 cm^−1^ band as a complementary indicator of IBU amorphization.

#### 4.2.4 Differential Scanning Calorimetry (DSC)

**Fig. 9:**
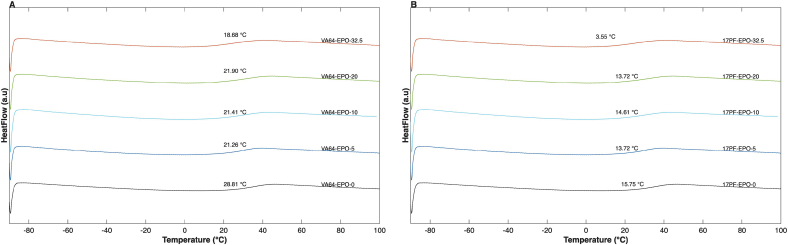
DSC second heating curves for: A) VA64-EPO EXT, B) 17PF-EPO EXT. The T_g_ values for each sample are depicted above each curve.

**Fig. 10:**
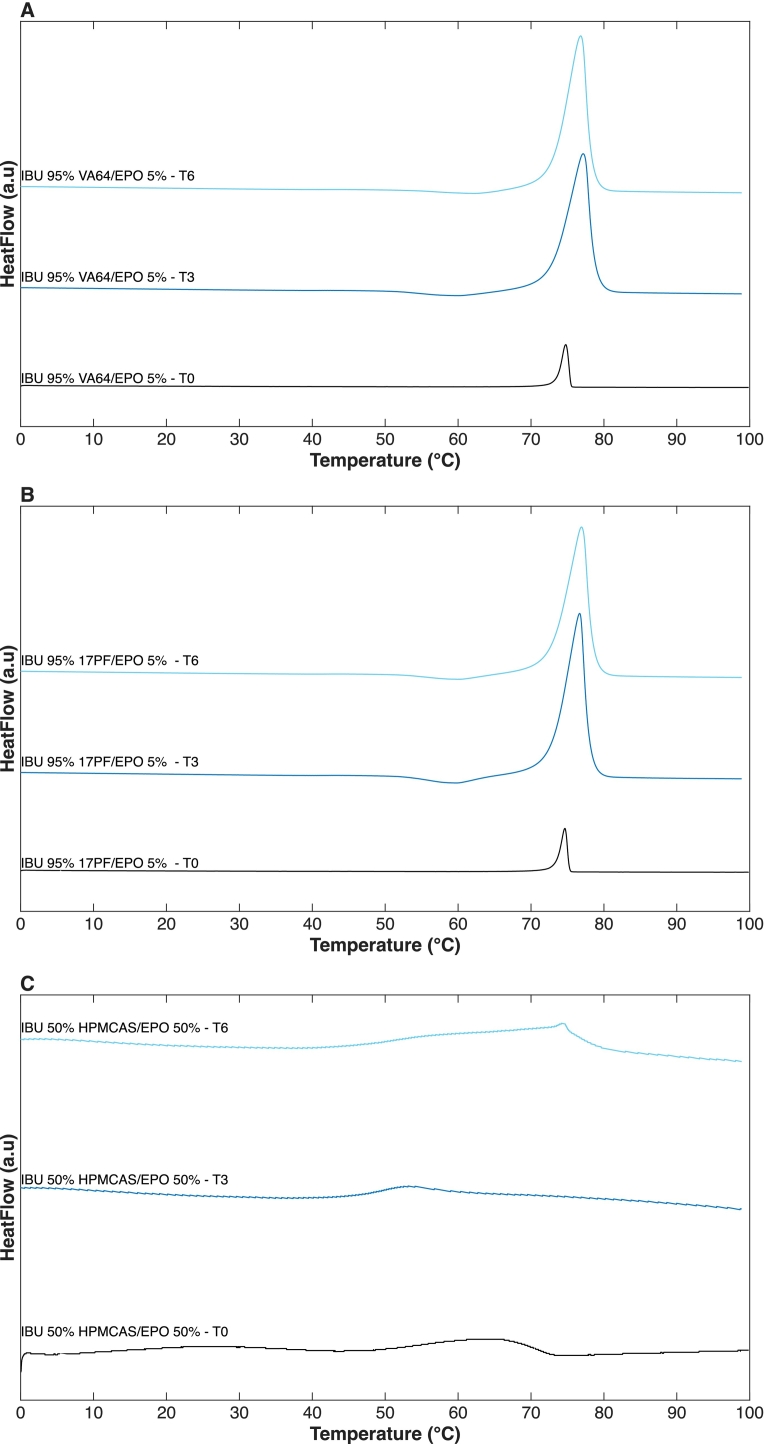
DSC first heating curves for high drug loading samples kept on DSC pans at T0, T3 and T6: A) VA64-EPO B) 17PF-EPO, C) HPMCAS-EPO. Curves show recrystallisation and melting events.

A single T_g_ (Fig. 9) was observed for all formulations during calorimetric analysis, confirming complete amorphization and miscibility (Ponnammal et al., 2018). The VA64-based blends T_g_ values were consistently higher than those of their 17PF counterparts (Fig. 9**A/B**). Considering that 17PF has a higher intrinsic T_g_ (132 °C), this corroborates that stronger API–polymer interactions occur in the 17PF systems, lowering overall T_g_ and supporting the interaction mechanisms discussed earlier. Considering EPO's low-T_g_ (52 °C), it should contribute to the plasticizing effect of blends. A measurable decrease in T_g_ was apparent when compared to the 5% EPO blend only for VA64. At intermediate EPO levels (5–20%), the T_g_ values remained largely comparable, indicating minimal additional influence within this concentration range. Only samples containing 32.5% EPO showed a steep T_g_ decrease, reflecting the same non-linear composition dependency observed in the MPD experiments in extrudates. A single T_g_ and no crystallinity peaks were evident in stability samples after three and six months (**Fig. S10 A-D**). In parallel, small-scale DSC pan samples prepared at drug loadings within the PC-SAFT-predicted unstable zone (IBU/VA64-EPO 95:5 wt%; IBU/17PF-EPO 95:5 wt%; IBU/HPMCAS-EPO 50:50 wt%) exhibited clear recrystallization followed by a melting event (Fig. 10 **A-C**), providing direct experimental evidence in support of PC-SAFT instability predictions. The presence of crystallinity in these samples is itself indicative of prior phase separation, as crystallization requires the formation of a drug-rich domain from which nucleation can proceed. This is especially significant given that IBU is considered a strong glass former (Blaabjerg et al., 2017), indicating that the observed instability is thermodynamically driven by exceeding the miscibility limit rather than reflecting any inherent crystallization tendency of the API.

#### 4.2.5 Non-sink dissolution

**Fig. 11:**
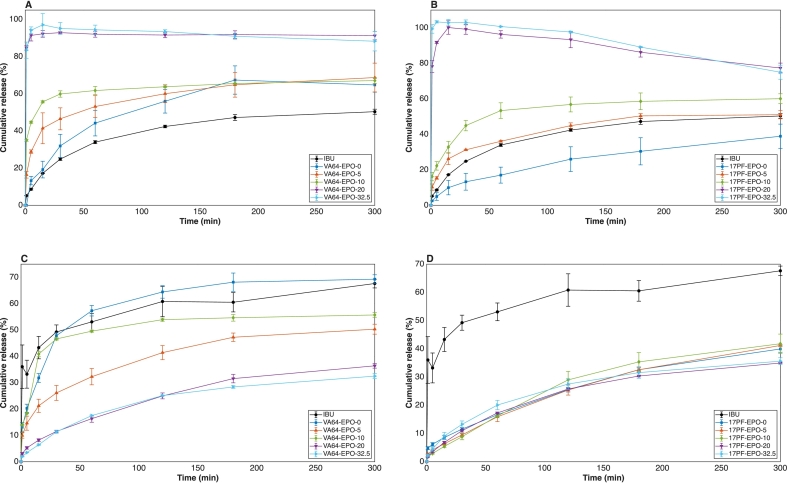
Cumulative IBU release in acidic conditions (pH 2) A) VA64-EPO, B)17PF-EPO and basic conditions (pH 6.8) C) VA64-EPO, D) 17PF-EPO (*n* = 3).

Considering the binary systems, VA64-EPO-0 showed only a modest improvement in cumulative release, whereas 17PF-EPO-0 displayed a slower and less efficient release profile (Fig. 11A**/B**). These differences reflect the complex interplay between API–polymer and polymer–water interactions during ASD dissolution. Strong API–polymer interactions can suppress water-induced amorphous–amorphous phase separation (AAPS), thereby maintaining a homogeneous amorphous phase (Kong et al., 2023). However, excessively strong interactions may hinder polymer dissolution by delaying chain hydration and disentanglement, particularly at high drug loadings (Que et al., 2021). In contrast, the milder IBU–VA64 interactions favor polymer hydration, facilitating faster drug release.

The incorporation of EPO introduced a distinct solubility enhancement mechanism. The proportional increase in both the initial burst and overall cumulative release observed at ≥20% EPO in both polymer blends can primarily be attributed to interactions between IBU and EPO's positively charged amino alkyl groups, which increase apparent solubility beyond amorphization alone (Saal et al., 2017; Xie and Taylor, 2017). Additionally, once EPO concentration exceeds its critical micellar concentration (CMC), micelles capable of solubilizing poorly water-soluble drugs such as IBU are formed (Lin et al., 2018). Finally, EPO may act as a microenvironmental pH modifier, locally lowering pH at the dissolving surface and further promoting drug ionization and dissolution (Almotairy et al., 2021). Together, these mechanisms drive the higher initial drug release and the generation of supersaturated conditions.

Beyond solubility enhancement, the ability to sustain supersaturation is equally critical for ASD performance. When drug concentration exceeds the amorphous solubility limit, the supersaturated system undergoes liquid-liquid phase separation (LLPS), generating drug-rich nanodroplets. These droplets can act as reservoirs that maintain elevated drug concentrations in the bulk phase but can also represent a crystallization precursor depending on drug loading, polymer characteristics, medium composition, etc. (Hermans et al., 2022). While EPO effectively promotes supersaturation, its capacity to inhibit recrystallization is limited, as API concentration tend to decline following the initial burst as already demonstrated elsewhere (Andrews et al., 2023; Lin et al., 2018; Tabriz et al., 2022; Xie and Taylor, 2017). On the other hand, the ability of poly-vinyl polymers to sustain supersaturation during dissolution is well established for acidic, poorly water-soluble APIs, with IBU and ketoprofen as examples (Browne et al., 2020; Chan et al., 2015; Tabriz et al., 2022; Ziaee et al., 2019). In VA64-based systems, the relatively hydrophobic character of VA64 favors API–polymer interactions in solution, maintaining supersaturation throughout 5 h with no evidence of precipitation (Fig. 11A). In contrast, the higher hydrophilicity of 17PF creates competition between polymer–water and polymer–API interactions, where preferential hydration of the polymer reduces effective API–polymer contact in solution, facilitating IBU crystallization (Janssens and Van Den Mooter, 2009) (Fig. 11B). These results highlight that generating and sustaining supersaturation requires the interplay between EPO and the PVP-based polymers. When such interplay is effective, it extends the limit of congruency (LoC) beyond the typical range of 5–20 wt% reported for conventional pharmaceutical polymers (Azevedo Costa, 2019; Benson et al., 2025; Danda et al., 2019; Tres et al., 2016) underscoring that polymer selection is a critical to ASD performance.

At pH 6.8, none of the ASDs significantly improved dissolution kinetics, as IBU is predominantly ionized (pKa = 4.9). However, differences in release profiles still provide mechanistic insight into API–polymer interactions under near-neutral conditions. For VA64-based systems (Fig. 11C), the progressive reduction in release with increasing EPO content reflects the limited solubility of EPO at near-neutral pH, where it remains largely insoluble and forms a diffusion barrier restricting drug release (Wang et al., 2022), consistent with a diffusion-controlled release mechanism (Sun et al., 2016). Conversely, for 17PF-based systems (Fig. 11D), release was independent of EPO content. Under these conditions, the ionized form of IBU may form stronger ion–dipole interactions with 17PF, further reducing drug mobility within the hydrated matrix and limiting diffusion into the dissolution medium. These results highlight that at near-neutral pH, dissolution behaviour is governed by polymer solubility, and that the ionisable character of EPO becomes a limiting rather than enabling factor.

## 5 Conclusions

ASDs remain one of the most effective formulation strategies for improving the performance of poorly soluble APIs. Building on previous work with binary IBU solid dispersions (De Castro et al., 2025), this study examined the influence of incorporating a third ionizable polymer on physical stability and dissolution performance. It was initially assumed that EPO would form additional API–polymer interactions, promoting further melting point depression and broadening stable and metastable composition ranges. However, no statistically significant differences were detected for VA64-EPO and 17PF-EPO systems, suggesting that IBU interaction sites are constrained by molecular size and conformation, consistent with observations in other ternary systems (Xie and Taylor, 2016). T_g_ measurements revealed sigmoidal profiles across all blends as API loading decreased from 90 to 20 wt%, with the BK model showing slightly improved predictive performance over conventional approaches, representing a suitable alternative for describing more complex free volume behaviour in ASD systems (Kalogeras, 2010; Kalogeras and Brostow, 2009; Pezzoli et al., 2018).

In-line UV–Vis spectroscopy successfully differentiated polymer types and detected compositional variations despite the high spectral similarity of raw data, with PCA identifying polymer type as the primary source of variability and EPO concentration as a secondary contributor. Samples clustered clearly according to composition, confirming the sensitivity of in-line UV–Vis to compositional differences at fixed drug loading and supporting predicted blend compatibility for ternary systems. Solid-state characterization confirmed full amorphization across all extrudates, evidenced by disappearance of the IBU crystalline peak at 778 cm^−1^ in FTIR and absence of crystalline peaks in XRD. Physical stability was confirmed in extrudate samples (35 wt%) stored at 25 °C/70% RH for up to six months, while small-scale DSC pan experiments at drug loadings within the PC-SAFT-predicted unstable zone (95 wt% IBU for VA64-EPO and 17PF-EPO; 50 wt% for HPMCAS-EPO) confirmed crystallization, providing direct experimental validation of the instability boundaries predicted by the model.

Within the experimental range, dissolution studies confirmed that stronger API–polymer interactions can be detrimental to drug release (Que et al., 2021), with EPO modulating release under acidic conditions and achieving complete dissolution at ≥20 wt% EPO. Despite improved release kinetics, 17PF-based systems showed limited recrystallisation inhibition, attributed to competing polymer–water interactions reducing precipitation inhibition capacity. Under basic conditions VA64 systems showed release inversely proportional to EPO content, consistent with insoluble barrier formation, while 17PF systems were EPO-independent, reflecting possible strong ion–dipole API–polymer interactions limiting drug diffusion.

Overall, the strong agreement between PC-SAFT predictions derived from binary k_ij_ and experimental outcomes across solid-state stability, extrusion processing, and dissolution performance supports a predict-first approach to ternary ASD formulation design, demonstrating that physics-based modeling can meaningfully guide polymer selection and drug loading optimization prior to experimentation.

## CRediT authorship contribution statement

**Matheus de Castro:** Writing – review & editing, Writing – original draft, Visualization, Validation, Software, Methodology, Investigation, Formal analysis, Data curation, Conceptualization. **Melissa Almeida:** Methodology, Investigation. **Christian Luebbert:** Writing – review & editing, Validation, Software, Methodology, Formal analysis. **Shadrack Joel Madu:** Resources, Methodology. **Jatin Khurana:** Writing – review & editing, Project administration, Funding acquisition. **Mark Evans:** Writing – review & editing, Project administration, Funding acquisition. **Matthew Leivers:** Writing – review & editing, Project administration, Funding acquisition. **Gabriel Araujo:** Writing – review & editing. **Mingzhong Li:** Writing – review & editing, Supervision, Resources. **Walkiria Schlindwein:** Writing – review & editing, Supervision, Project administration, Funding acquisition, Conceptualization.

## Declaration of competing interest

The authors declare the following financial interests/personal relationships which may be considered as potential competing interests.

Matheus de Castro reports financial support was provided by Reckitt Benckiser LLC. If there are other authors, they declare that they have no known competing financial interests or personal relationships that could have appeared to influence the work reported in this paper.

## Data Availability

Data will be made available on request.

## Appendix A Supplementary data

Supplementary material 1: Fig. S1. DSC thermograms of IBU–polymer blends at varying API–polymer compositions, showing glass transition temperatures determined at a heating rate of 1°C/min. A) IBU–VA64–EPO, B) IBU–17PF–EPO, C) IBU–HPMCAS–EPO

Supplementary material 2: Fig. S2. Melting end set temperatures of binary and ternary blends determined at a heating rate of 1°C/min. A) IBU–VA64–EPO, B) IBU–17PF–EPO, C) IBU–HPMCAS–EPO

Supplementary material 3: Fig. S3. Glass transition temperatures (Tg) of binary and ternary blends determined at a heating rate of 1°C/min. A) IBU–VA64–EPO, B) IBU–17PF–EPO, C) IBU–HPMCAS–EPO

Supplementary material 4: Fig. S4. FTIR zoomed view at 778 cm^-1^ (IBU crystalline peak region) for extrudates (EXT) and physical mixtures (PM) obtained immediately after extrusion (T0). A) IBU–VA64–EPO EXT, B) IBU–VA64–EPO PM, C) IBU–17PF–EPO EXT, D) IBU–17PF–EPO PM

Supplementary material 5: Fig. S5. FTIR spectra of extrudate samples following three months of storage (T3) at 25°C/70% RH. A) IBU–VA64–EPO full spectra, B) IBU–VA64–EPO zoomed at 778 cm^-1^, C) IBU–17PF–EPO full spectra, D) IBU–17PF–EPO zoomed at 778 cm^-1^

Supplementary material 6: Fig. S6. FTIR spectra of extrudate samples following six months of storage (T6) at 25°C/70% RH. A) IBU–VA64–EPO full spectra, B) IBU–VA64–EPO zoomed at 778 cm^-1^, C) IBU–17PF–EPO full spectra, D) IBU–17PF–EPO zoomed at 778 cm^-1^

Supplementary material 7: Fig S7. Polymers PXRD diffractogram showing amorphous pattern

Supplementary material 8: Fig. S8. PXRD diffractograms of extrudate samples following three months of storage (T3) at 25°C/70% RH. A) IBU–VA64–EPO, B) IBU–17PF–EPO

Supplementary material 9: Fig. S9. PXRD diffractograms of extrudate samples following six months of storage (T6) at 25°C/70% RH. A) IBU–VA64–EPO, B) IBU–17PF–EPO

Supplementary material 10: Fig. S10. DSC thermograms of extrudate samples stored at 25°C/70% RH at three months (T3): A) IBU–VA64–EPO, B) IBU–17PF–EPO; and six months (T6): C) IBU–VA64–EPO, D) IBU–17PF–EPO

Supplementary material 11: Table S1. Tg modelling parameters for GT, Kwei and BK models
