# Supplementary figures and images for: Hot-melt extruded ibuprofen ternary solid dispersions using in-line UV–Vis: Impact of an ionizable polymer on thermodynamics and dissolution
Source: Int J Pharm X. 2026 Apr 6;11:100536. doi: 10.1016/j.ijpx.2026.100536 (PMC13091325; doi:10.1016/j.ijpx.2026.100536)

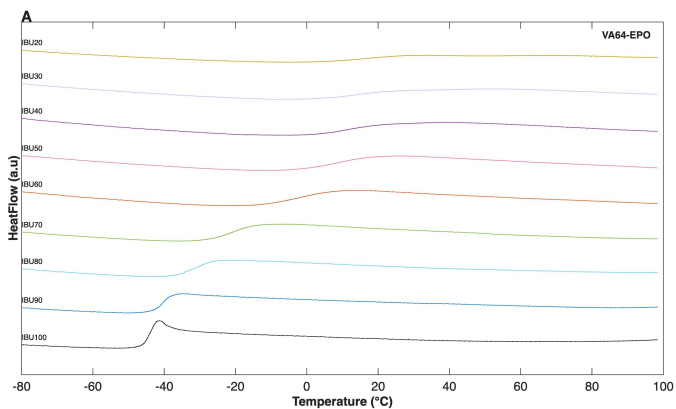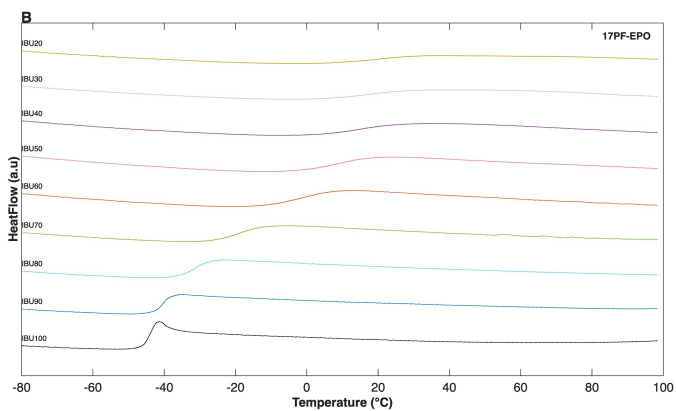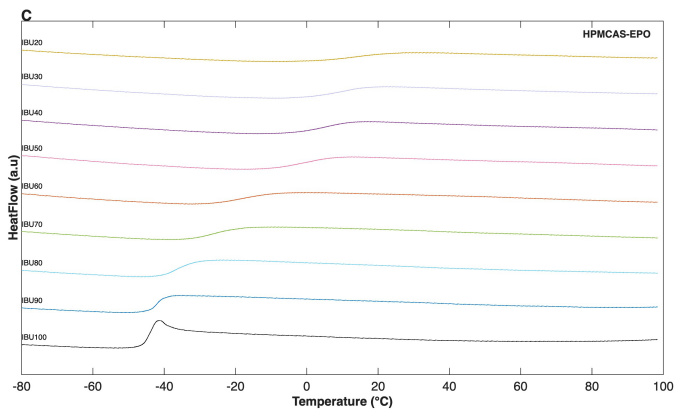

Supplement: Supplementary file 1 — Supplementary material 1: Fig. S1. DSC thermograms of IBU–polymer blends at varying API–polymer compositions, showing glass transition temperatures determined at a heating rate of 1°C/min. A) IBU–VA64–EPO, B) IBU–17PF–EPO, C) IBU–HPMCAS–EPO [file mmc1.pdf]

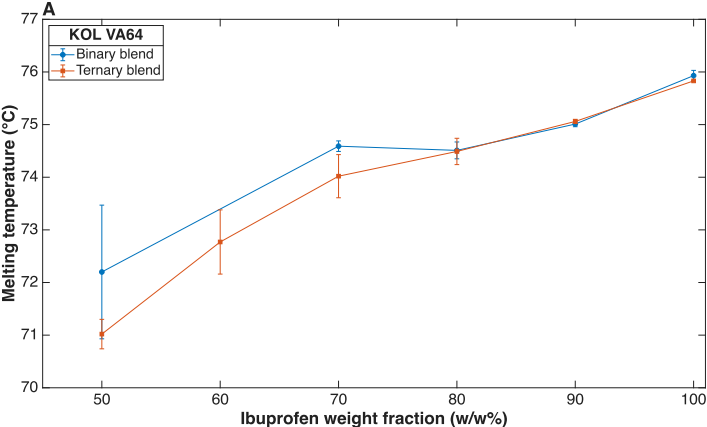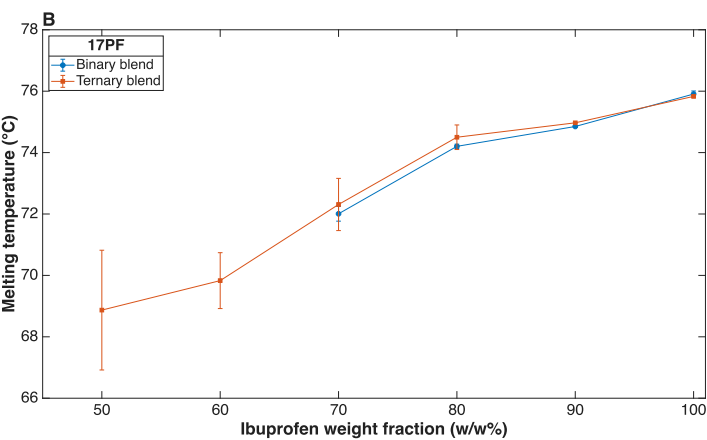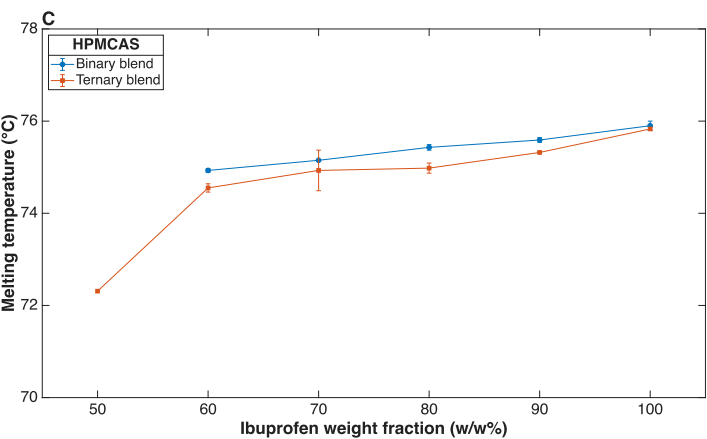

Supplement: Supplementary file 2 — Supplementary material 2: Fig. S2. Melting end set temperatures of binary and ternary blends determined at a heating rate of 1°C/min. A) IBU–VA64–EPO, B) IBU–17PF–EPO, C) IBU–HPMCAS–EPO [file mmc2.pdf]

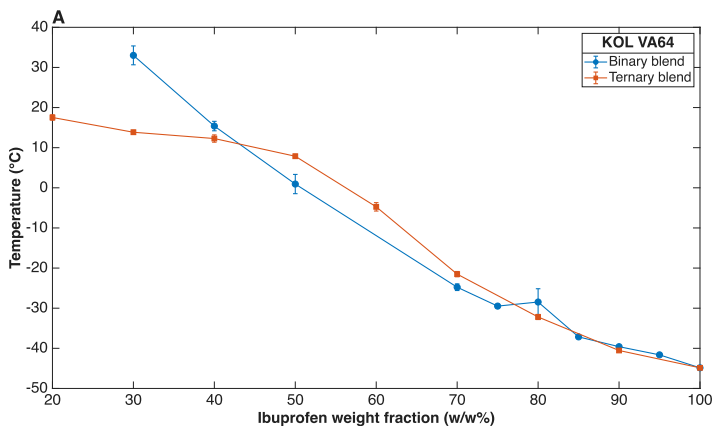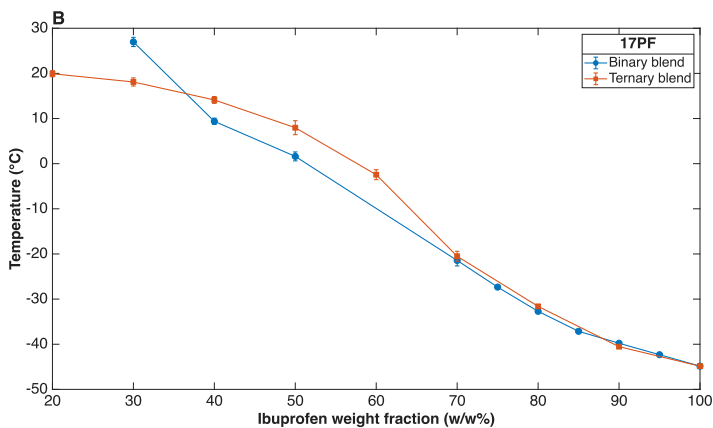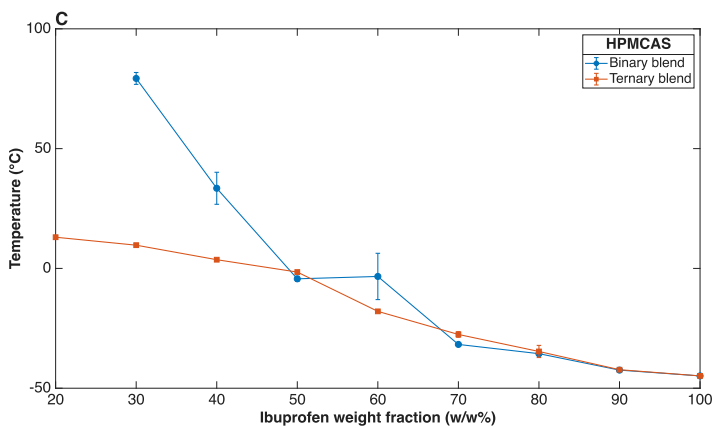

Supplement: Supplementary file 3 — Supplementary material 3: Fig. S3. Glass transition temperatures (Tg) of binary and ternary blends determined at a heating rate of 1°C/min. A) IBU–VA64–EPO, B) IBU–17PF–EPO, C) IBU–HPMCAS–EPO [file mmc3.pdf]

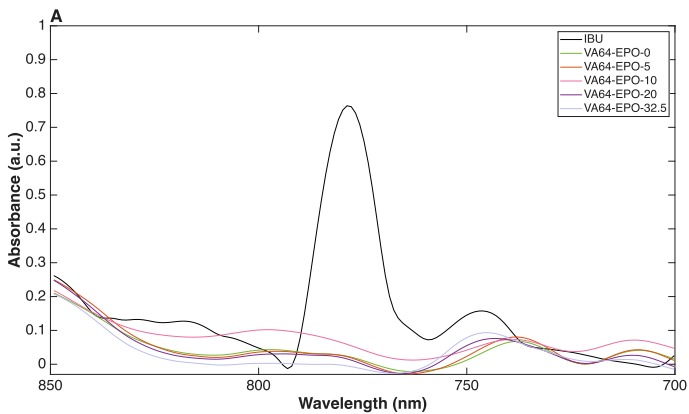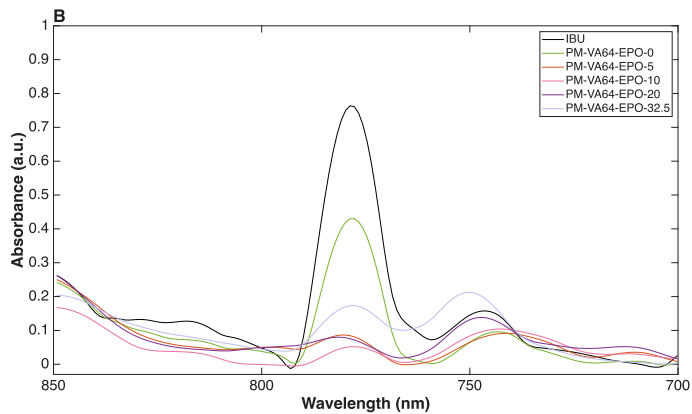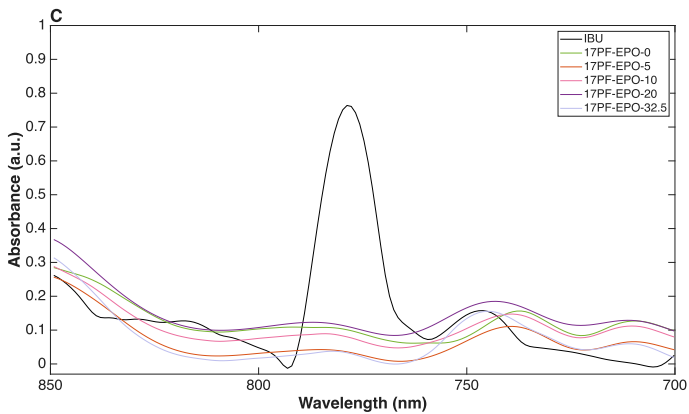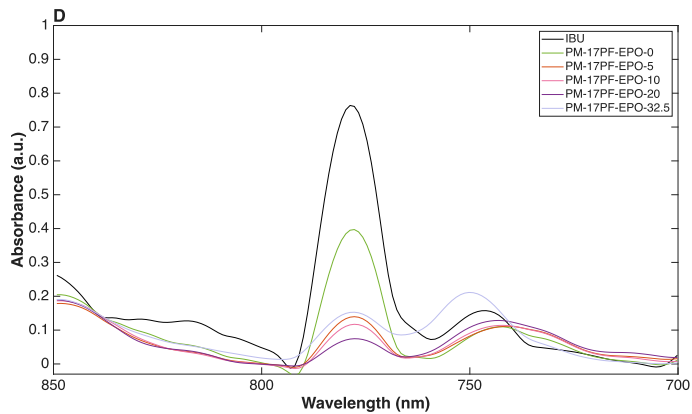

Supplement: Supplementary file 4 — Supplementary material 4: Fig. S4. FTIR zoomed view at 778 cm-1 (IBU crystalline peak region) for extrudates (EXT) and physical mixtures (PM) obtained immediately after extrusion (T0). A) IBU–VA64–EPO EXT, B) IBU–VA64–EPO PM, C) IBU–17PF–EPO EXT, D) IBU–17PF–EPO PM [file mmc4.pdf]

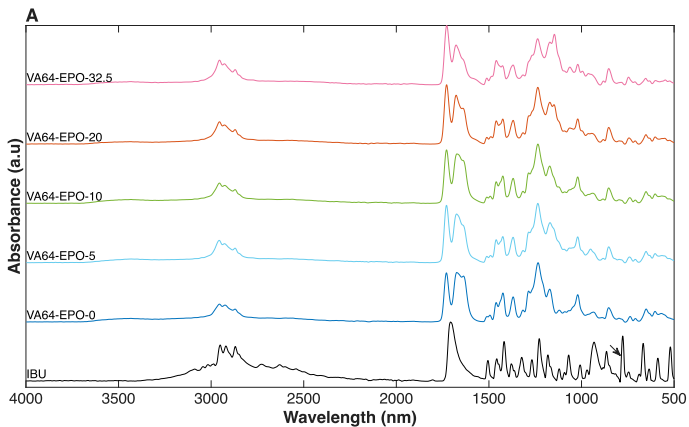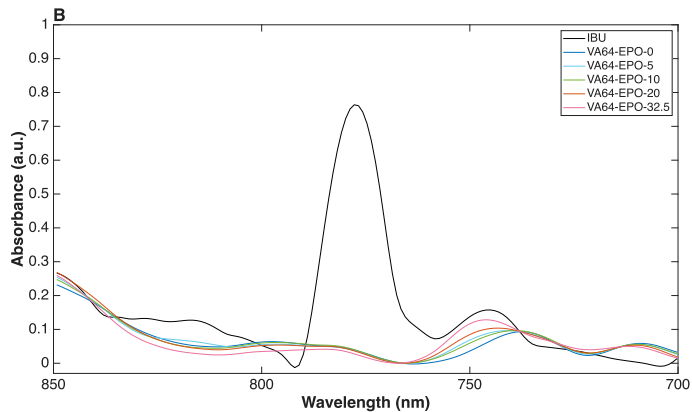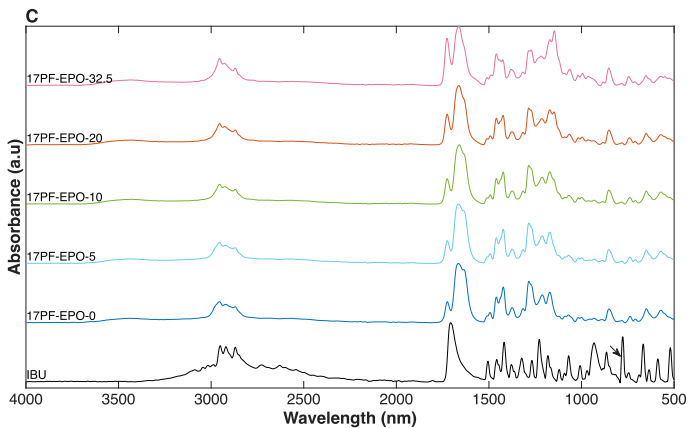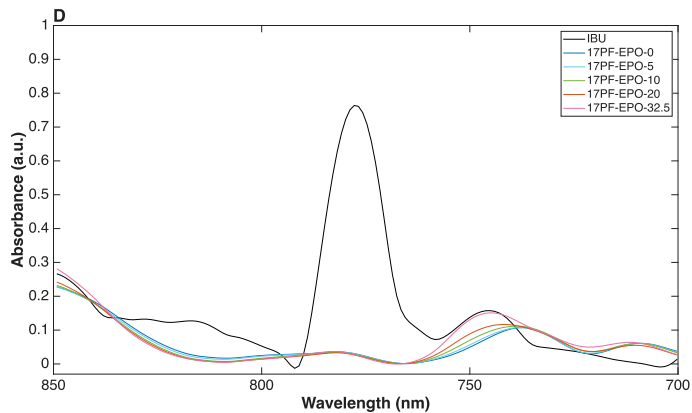

Supplement: Supplementary file 5 — Supplementary material 5: Fig. S5. FTIR spectra of extrudate samples following three months of storage (T3) at 25°C/70% RH. A) IBU–VA64–EPO full spectra, B) IBU–VA64–EPO zoomed at 778 cm-1, C) IBU–17PF–EPO full spectra, D) IBU–17PF–EPO zoomed at 778 cm-1 [file mmc5.pdf]

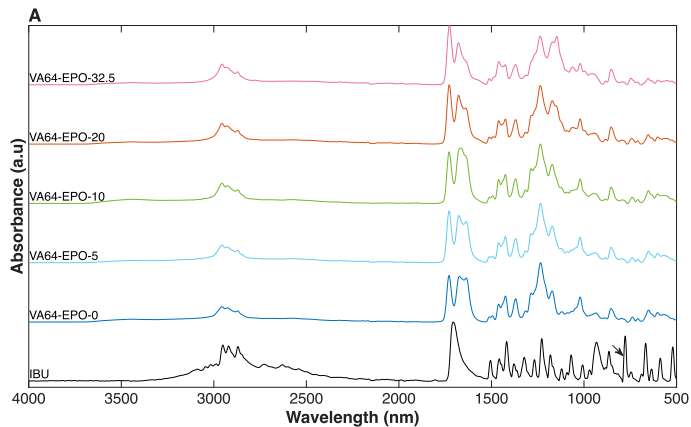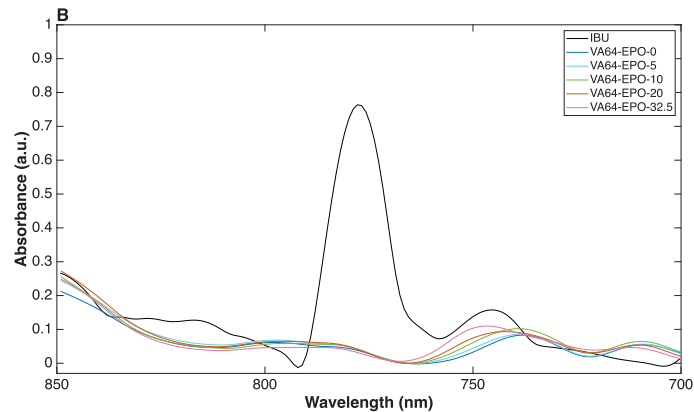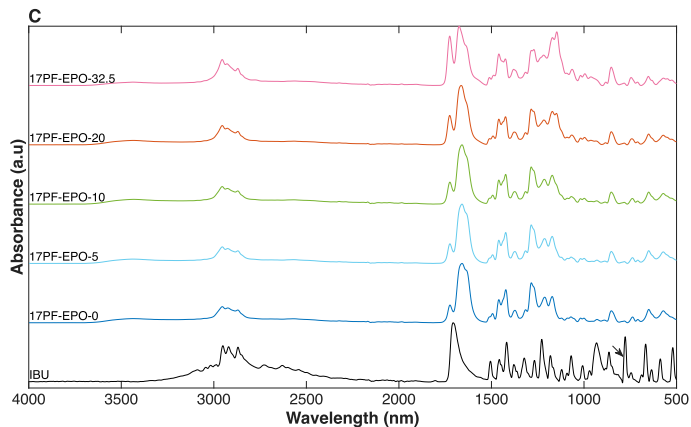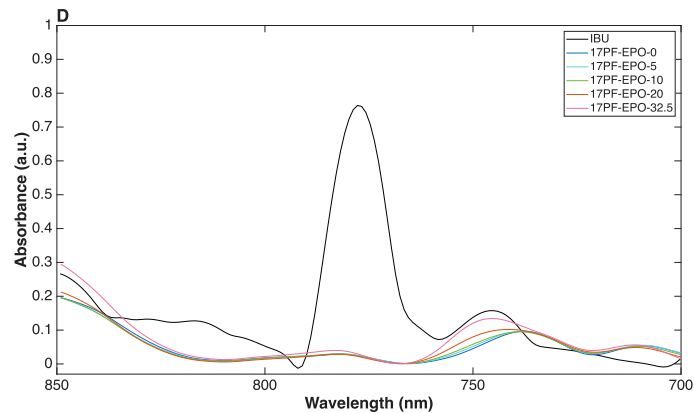

Supplement: Supplementary file 6 — Supplementary material 6: Fig. S6. FTIR spectra of extrudate samples following six months of storage (T6) at 25°C/70% RH. A) IBU–VA64–EPO full spectra, B) IBU–VA64–EPO zoomed at 778 cm-1, C) IBU–17PF–EPO full spectra, D) IBU–17PF–EPO zoomed at 778 cm-1 [file mmc6.pdf]

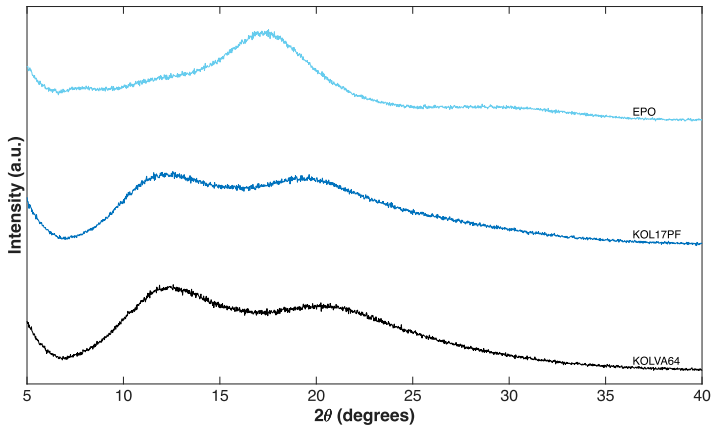

Supplement: Supplementary file 7 — Supplementary material 7: Fig S7. Polymers PXRD diffractogram showing amorphous pattern [file mmc7.pdf]

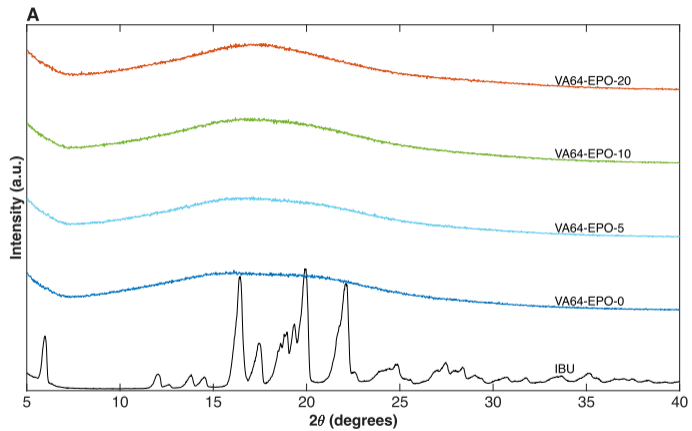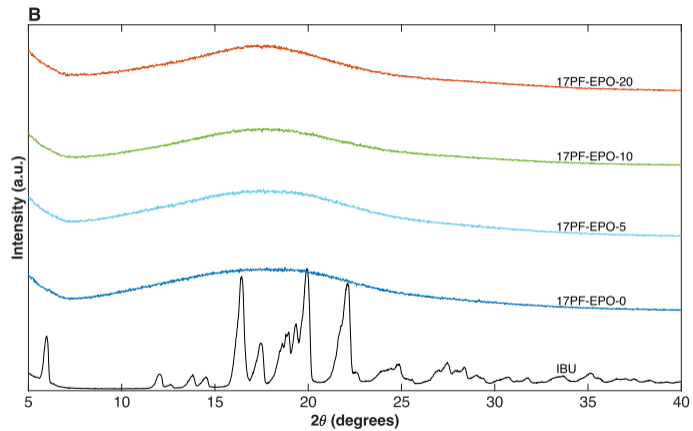

Supplement: Supplementary file 8 — Supplementary material 8: Fig. S8. PXRD diffractograms of extrudate samples following three months of storage (T3) at 25°C/70% RH. A) IBU–VA64–EPO, B) IBU–17PF–EPO [file mmc8.pdf]

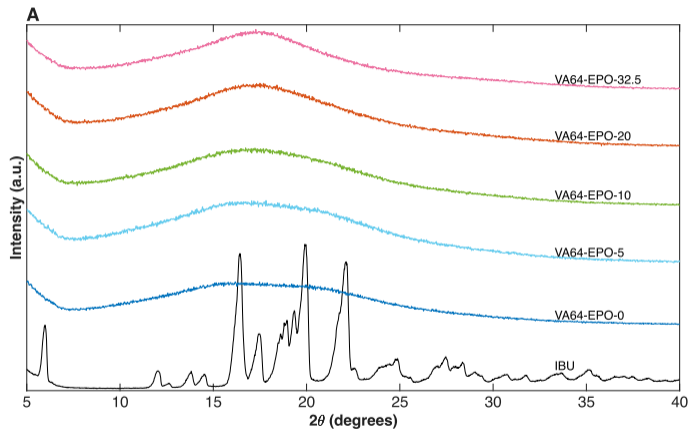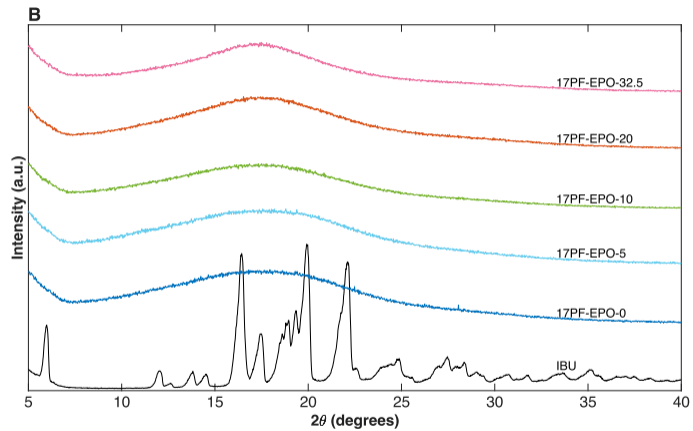

Supplement: Supplementary file 9 — Supplementary material 9: Fig. S9. PXRD diffractograms of extrudate samples following six months of storage (T6) at 25°C/70% RH. A) IBU–VA64–EPO, B) IBU–17PF–EPO [file mmc9.pdf]

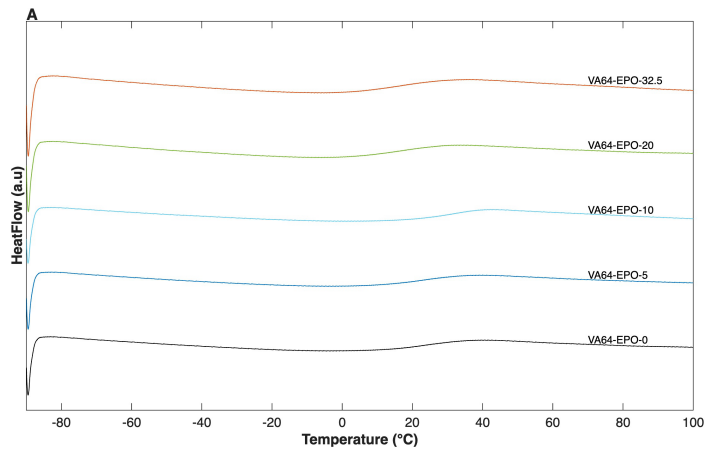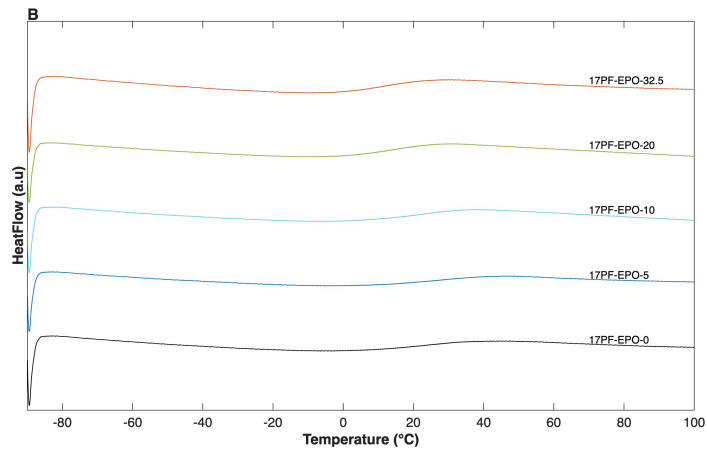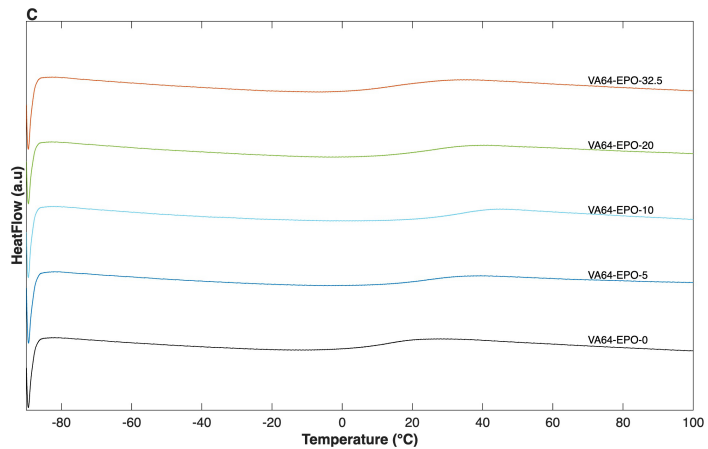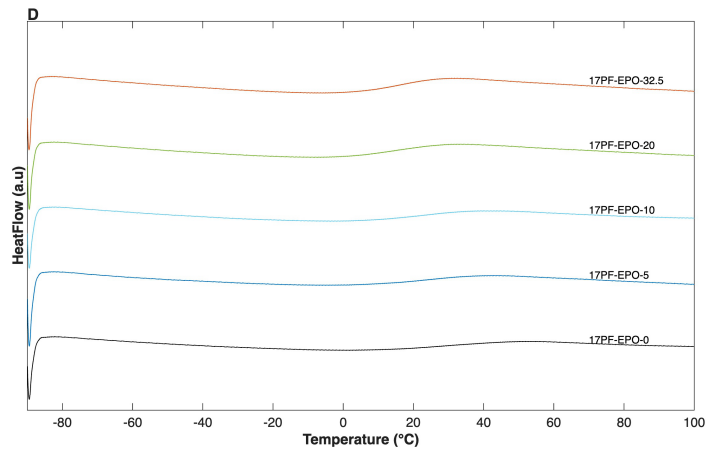

Supplement: Supplementary file 10 — Supplementary material 10: Fig. S10. DSC thermograms of extrudate samples stored at 25°C/70% RH at three months (T3): A) IBU–VA64–EPO, B) IBU–17PF–EPO; and six months (T6): C) IBU–VA64–EPO, D) IBU–17PF–EPO [file mmc10.pdf]
