# Supplementary material for: Hot-melt extruded ibuprofen ternary solid dispersions using in-line UV–Vis: Impact of an ionizable polymer on thermodynamics and dissolution
Source: Int J Pharm X. 2026 Apr 6;11:100536. doi: 10.1016/j.ijpx.2026.100536 (PMC13091325; doi:10.1016/j.ijpx.2026.100536)
Supplement: Supplementary file 11 — Supplementary material 11: Table S1. Tg modelling parameters for GT, Kwei and BK models [file mmc11.docx]

Hot-Melt Extruded Ibuprofen Ternary Solid Dispersions using In-Line UV-Vis: Impact of an Ionizable Polymer on Thermodynamics and Dissolution

| **Matheus de Castro**^1^**; Melissa Almeida^2^; Christian Luebbert^3^; Shadrack Joel Madu^1^; Jatin Khurana^4^, Mark Evans^4^, Matthew Leivers^4^, Gabriel Araujo^2^, Mingzhong Li^1^, Walkiria Schlindwein^1*^** |
| --- |
| ^1^ Leicester School of Pharmacy, De Montfort University, Leicester LE1 9BH, United Kingdom.  ^2^ Department of Pharmacy, School of Pharmaceutical Sciences, University of São Paulo, São Paulo, SP, Brazil.  ^3^ amofor GmbH, Otto-Hahn-Str. 15, 44227 Dortmund, Germany  ^4^ Reckitt Benckiser, Dansom Lane, Hull HU8 7DS, United Kingdom. |
|  |

**SUPPLEMENTARY INFORMATION**

**Table S1.** T_g_ modelling parameters for GT, Kwei and BK models.

| **System** | **Model** | **Parameters** | **R²** | **ARD (%)** | **AARD (%)** |
| --- | --- | --- | --- | --- | --- |
| **IBU-VA64-EPO** | GT | K_1_ = −1.035; K_2_ = 3.552 | 0.9560 | +0.39 | 1.65 |
|  | Kwei | q₁ = 0.1533; q_2_ = 0.1421 | 0.9506 | +0.15 | 1.69 |
|  | BK | a₀ = −53.67; a₁ = 124.4; a₂ = −291.3 | 0.9890 | −0.07 | 0.76 |
| **IBU-17PF-EPO** | GT | K_1_ = -0.5724; K_2_ = 3.034 | 0.9616 | +0.40 | 1.60 |
|  | Kwei | q_1_ = 0.1612; q_2_ = 0.1421 | 0.9571 | +0.17 | 1.62 |
|  | BK | a₀ = −70.0; a₁ = 144.2; a₂ = −309.2 | 0.9881 | −0.07 | 0.79 |
| **IBU-HPMCAS-EPO** | GT | K_1_= −0.6025; K_2_ = 2.367 | 0.9583 | +0.37 | 1.42 |
|  | Kwei | q_1_ = 0.1353; q_2_ = 0.1212 | 0.9568 | +0.48 | 1.50 |
|  | BK | a₀ = −103.6; a₁ = 136.8; a₂ = −265.2 | 0.9696 | −0.06 | 1.28 |

**Fig. S1.** DSC thermograms of IBU–polymer blends at varying API–polymer compositions, showing glass transition temperatures determined at a heating rate of 1°C/min. A) IBU–VA64–EPO, B) IBU–17PF–EPO, C) IBU–HPMCAS–EPO.

**Fig. S2.** Melting end set temperatures of binary and ternary blends determined at a heating rate of 1°C/min. A) IBU–VA64–EPO, B) IBU–17PF–EPO, C) IBU–HPMCAS–EPO.

**Fig. S3.** Glass transition temperatures (T_g_) of binary and ternary blends determined at a heating rate of 1°C/min. A) IBU–VA64–EPO, B) IBU–17PF–EPO, C) IBU–HPMCAS–EPO.

**Fig. S4.** FTIR zoomed view at 778 cm⁻¹ (IBU crystalline peak region) for extrudates (EXT) and physical mixtures (PM) obtained immediately after extrusion (T0). A) IBU–VA64–EPO EXT, B) IBU–VA64–EPO PM, C) IBU–17PF–EPO EXT, D) IBU–17PF–EPO PM.

**Fig. S5.** FTIR spectra of extrudate samples following three months of storage (T3) at 25°C/70% RH. A) IBU–VA64–EPO full spectra, B) IBU–VA64–EPO zoomed at 778 cm⁻¹, C) IBU–17PF–EPO full spectra, D) IBU–17PF–EPO zoomed at 778 cm⁻¹.

**Fig. S6.** FTIR spectra of extrudate samples following six months of storage (T6) at 25°C/70% RH. A) IBU–VA64–EPO full spectra, B) IBU–VA64–EPO zoomed at 778 cm⁻¹, C) IBU–17PF–EPO full spectra, D) IBU–17PF–EPO zoomed at 778 cm⁻¹.

**Fig S7.** Polymers PXRD diffractogram showing amorphous pattern.

**Fig. S8.** PXRD diffractograms of extrudate samples following three months of storage (T3) at 25°C/70% RH. A) IBU–VA64–EPO, B) IBU–17PF–EPO.

**Fig. S9.** PXRD diffractograms of extrudate samples following six months of storage (T6) at 25°C/70% RH. A) IBU–VA64–EPO, B) IBU–17PF–EPO.

**Fig. S10.** DSC thermograms of extrudate samples stored at 25°C/70% RH at three months (T3): A) IBU–VA64–EPO, B) IBU–17PF–EPO; and six months (T6): C) IBU–VA64–EPO, D) IBU–17PF–EPO.
